# Amplicon sequencing for the quantification of spoilage microbiota in complex foods including bacterial spores

**DOI:** 10.1186/s40168-015-0096-3

**Published:** 2015-07-27

**Authors:** Paulo de Boer, Martien Caspers, Jan-Willem Sanders, Robèr Kemperman, Janneke Wijman, Gijs Lommerse, Guus Roeselers, Roy Montijn, Tjakko Abee, Remco Kort

**Affiliations:** TI Food and Nutrition, Wageningen, The Netherlands; TNO Microbiology and Systems Biology, Utrechtseweg 48, 3704 HE Zeist, The Netherlands; Unilever R&D, Vlaardingen, The Netherlands; Corbion, Gorinchem, The Netherlands; Laboratory of Food Microbiology, Wageningen University and Research Centre, Wageningen, The Netherlands; Molecular Cell Physiology, VU University Amsterdam, Amsterdam, The Netherlands

**Keywords:** Spoilage microbiota, Lactic acid bacteria, Bacterial spores, Amplicon sequencing, DNA extraction, Quantification

## Abstract

**Background:**

Spoilage of food products is frequently caused by bacterial spores and lactic acid bacteria. Identification of these organisms by classic cultivation methods is limited by their ability to form colonies on nutrient agar plates. In this study, we adapted and optimized 16S rRNA amplicon sequencing for quantification of bacterial spores in a canned food matrix and for monitoring the outgrowth of spoilage microbiota in a ready-to-eat food matrix.

**Results:**

The detection limit of bar-coded 16S rRNA amplicon sequencing was determined for the number of bacterial spores in a canned food matrix. Analysis of samples from a canned food matrix spiked with a mixture of equinumerous spores from the thermophiles, *Geobacillus stearothermophilus* and *Geobacillus thermoglucosidans*, and the mesophiles, *Bacillus sporothermodurans*, *Bacillus cereus*, and *Bacillus subtilis*, led to the detection of these spores with an average limit of 2 × 10^2^ spores ml^−1^. The data were normalized by setting the number of sequences resulting from DNA of an inactivated bacterial species, present in the matrix at the same concentration in all samples, to a fixed value for quantitative sample-to-sample comparisons. The 16S rRNA amplicon sequencing method was also employed to monitor population dynamics in a ready-to-eat rice meal, incubated over a period of 12 days at 7 °C. The most predominant outgrowth was observed by the genera *Leuconostoc*, *Bacillus*, and *Paenibacillus*. Analysis of meals pre-treated with weak acids showed inhibition of outgrowth of these three genera. The specificity of the amplicon synthesis was improved by the design of oligonucleotides that minimize the amplification of 16S rRNA genes from chloroplasts originating from plant-based material present in the food.

**Conclusion:**

This study shows that the composition of complex spoilage populations, including bacterial spores, can be monitored in complex food matrices by bar-coded amplicon sequencing in a quantitative manner. In order to allow sample-to-sample comparisons, normalizations based on background DNA are described. This method offers a solution for the identification and quantification of spoilage microbiota, which cannot be cultivated under standard laboratory conditions. The study indicates variable detection limits among species of bacterial spores resulting from differences in DNA extraction efficiencies.

**Electronic supplementary material:**

The online version of this article (doi:10.1186/s40168-015-0096-3) contains supplementary material, which is available to authorized users.

## Background

Microbial food spoilage results from metabolic processes that lead to the production of off-smelling flavors or textural changes and renders food unacceptable for human consumption. It is by far the most common cause for food losses. It has been estimated that 25 % of all foods produced globally is lost due to microbial spoilage, see [1, 2] and references herein. In this study, we applied the 16S rRNA amplicon pyrosequencing method for the evaluation of microbial spoilage in complex foods, including a ready-to-eat (RTE) meal as well as processed canned food. These are very different in nature with regard to the spoilage process. In the first case, outgrowth of spoilage microorganisms is limited by refrigeration and preservatives, and in the second case, heat processing during manufacturing limits the spoilage problem to outgrowth of bacterial spores that are able to survive the heat regimes applied.

Characterization and enumeration of microbial species on nutrient agar plates is still the standard method to evaluate the cause of microbial spoilage and to find clues for the design of preservation strategies. However, typing and enumeration of strains or communities of species by classic cultivation methods is laborious, time-consuming, and biased by selective germination and outgrowth conditions on nutrient agar plates [3, 4]. Microbial spoilage can be regarded as a complex ecological process, which can involve the presence of multiple species with specific niches and metabolic dependencies; this may be easily overlooked in case of cultivation on nutrient agar plates with a relatively rich and homogenous environment, where a single or a small number of microbial species easily dominate [2].

A number of cultivation-independent methods have been developed that identify the most abundant microbial species in the spoilage population *at the time of sampling* by isolation and characterization of their DNA molecules. However, these methods are not suited for the quantification of the species in a population. Until recently, most of these cultivation-independent studies on typing of bacterial populations were based on PCR-DGGE [5] and microarray technology [6–8]. With the arrival of next-generation sequencing methods, such as pyrosequencing of bar-coded 16S rRNA amplicons [9, 10], it is now feasible to analyze microbial populations in multiple samples in parallel up to the genus and even the species level. This methodology has been applied in a number of cases to characterize spoilage microbiota [11, 12], as reviewed recently by Danilo Ercolini [13]. With the further decrease of DNA sequencing costs, this appears a promising method for future microbiota analyses in complex foods.

The aims linked to the bar-coded 16S rRNA amplicon sequencing for evaluation of spoilage microbiota included methodology development for (i) reproducible and efficient extraction methodology DNA from complex food matrices and (ii) sample-to-sample comparisons of microbial compositions by means of normalization based on background or spiked DNA. In addition, we aim to monitor and interpret differences of amplicon read counts and colony-forming unit (CFU) counts by application of both analyses to the same samples.

In this study, we addressed these challenges associated with bar-coded 16S rRNA amplicon sequencing applied to two types of food matrices. The first part of the study was aimed at establishing the limit of bar-coded amplicon sequencing for detection of bacterial spores and their quantification in a processed canned food matrix. The population composition after incubation at moderate and high temperatures was investigated, in order to observe specific outgrowth of spores from selected mesophilic *Bacillus* and thermophilic *Geobacillus* species. In the second part of the study, bacterial outgrowth of the spoilage microbiota was analyzed in a ready-to-eat meal stored at 7 °C in the absence and presence of weak organic acids, which are commonly used food preservatives (reviewed in [14]). Limitations of this cultivation-independent methodology and differences in the outcome of CFU enumeration have been evaluated by application of both methods to the same samples. This study shows that bar-coded amplicon sequencing can be applied in a quantitative manner at low detection limit in food matrices.

## Results

### Detection of spores in a canned food matrix by colony enumeration

**Fig. 1:**
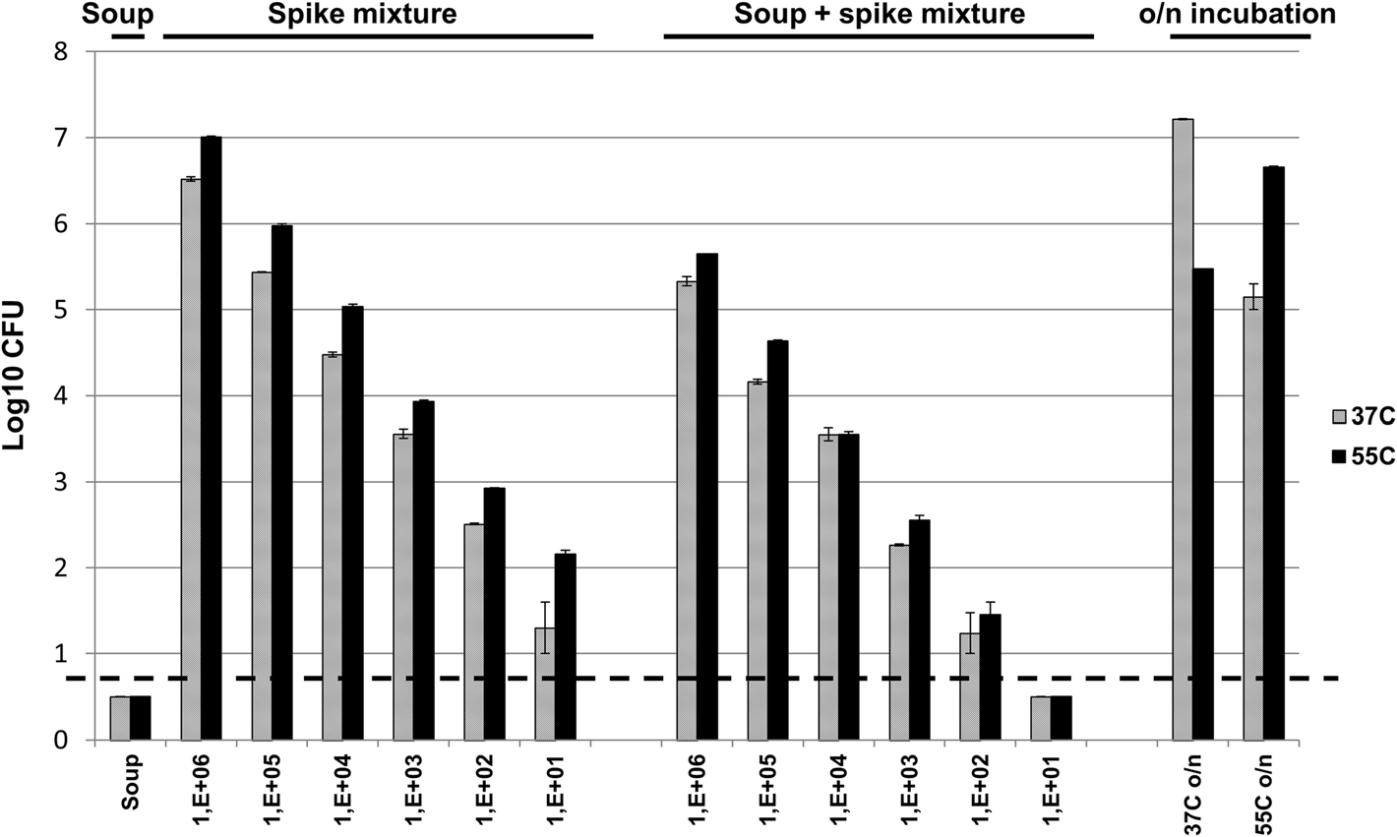
Viable counts of a spore spoilage model for a canned food matrix. Colony-forming units (CFUs) of pure canned food (soup), the pure spore mixture of five species diluted in physiological salt (spike mixture), canned food inoculated with the spike mixture in diluted series (soup + spike mixture), and canned food spiked with 1 × 10^6^ spores and incubated at 37 or 55 °C (o/n incubation) are shown. CFU counts were determined on TSA plates aerobically incubated at 37 °C (*hatched bars*) or 55 °C (*black bars*). *Error bars* represent duplicates of separate canned food, spore, or canned food + spore batches. The *dashed black line* (logCFU = 1) indicates the detection limit: *bars* below this limit represent samples where no growth was observed

The “spike” spore mixture consisting of three mesophilic species, *B. subtilis* A163, *B. cereus* TNO 02.0143, and *B. sporothermodurans* IC4, two thermophilic species, *G. thermoglucosidans* TNO 09.020 and *G. stearothermophilus* ATCC 7953, and tenfold dilutions of these five strains were mixed into creamy mushroom soup. CFU counts of the spore suspensions in the canned food or in physiological salt solution showed a near linear logarithmic CFU decrease (Fig. 1). However, CFU counts were approximately 1 log unit lower in the canned food when compared to dilution in physiological salt solution, indicating that the canned food matrix suppresses CFU formation from the spores. In non-inoculated canned food, no CFUs were observed (less than 10 CFU/ml). The effect of incubation at 37 or 55 °C was investigated on canned food aliquots inoculated with the highest spore concentration (10^6^ spores per species per ml). As expected, outgrowth of mesophiles was observed after o/n incubation at 37 °C and of thermophiles after incubation at 55 °C (Fig. 1).

### Filtering of sequence reads and normalization

After DNA extraction from 180 samples, 16S rRNA amplicons were obtained and sequenced by bar-coded amplicon sequencing using the Roche 454 platform, leading to an average of 6171 ± 3367 (SD) raw sequences per sample. A total of 15 samples gave insufficient reads (<1000) and were discarded in further analysis. Processing with the Mothur pipeline yielded 6203 ± 2980 (SD) reads for the canned food or spike samples and 4091 ± 1364 (SD) reads for the RTE rice meal samples. After assigning operational taxonomic units (OTUs), the varying total sequences of the ready-to-eat meal were normalized by setting the total number of sequences for each sample to 10,000 reads. More details regarding the processing of the sequence data have been provided in Additional file 1.

### Detection of bacterial spores in a canned food matrix by amplicon sequencing

**Fig. 2:**
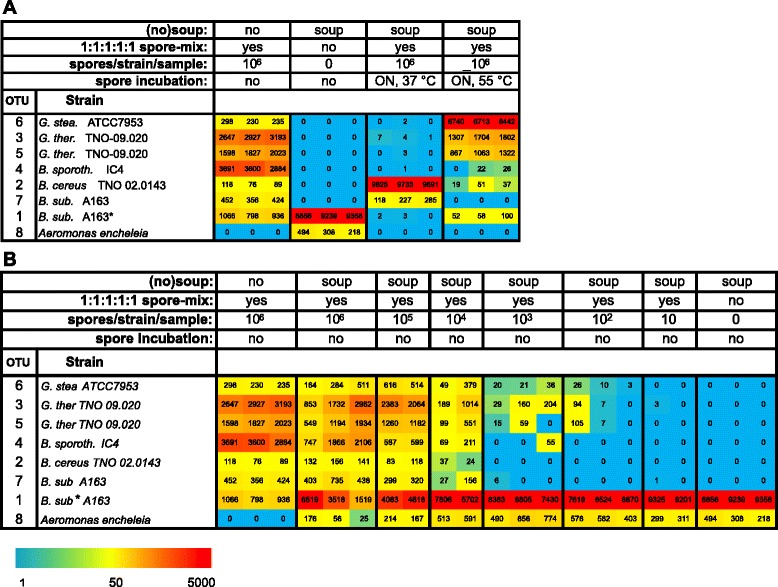
Amplicon sequencing-based microbial typing and semi-quantitative detection of bacterial spores in canned food. The *heat map* shows the number of normalized reads per sample per unique sequence (below the heat map the legend of the color code is provided). For clarity, the actual numbers of normalized reads per sample are also shown. **a** Pure spores, pure canned food, and the effect of o/n outgrowth in canned food at 37 or 55 °C. **b** Pure spores and serial dilutions of spores in canned food matrix. Note that for the 10^4^ and 10^5^, only two numbers are shown due to technical failure of the third samples

In order to evaluate the identity and quantity of OTUs of the spore suspensions of five bacterial strains, samples were analyzed. Calculations based on 100 % identity (0 % difference = 1 unique sequence per OTU) resulted in a total of seven OTUs, indicating that multiple OTUs corresponded to one species. Careful analysis indicated that both *G. thermoglucosidans* TNO09-020 and *B. subtilis* A163 are represented by two OTUs (Fig. 2a). Despite the equal number of added spores in the spike (based on microscopic counting), a strong unequal distribution of OTUs was observed (Fig. 2a).

Sequence analysis of the creamy mushroom soup without inoculum shows the total absence of sequences belonging to four of the five species of the spike (Fig. 2a). The majority of the sequences detected in the canned food did however belong to OTU 1, which was also observed for *B. subtilis* A163. Since no detectable CFUs were observed for the canned food, this indicated the presence of inactivated spores or cells of *B. subtilis* A163—or a closely related strain—in the canned food without inoculum. Note that also other bacterial species were observed, which did not correspond to the spike species, in the canned food, but in much lower numbers than OTU 1 (Additional file 2). One example is *Aeromonas encheleia* (Fig. 2a), which was used in later analysis for normalization purposes.

Amplicon sequence analysis of the inoculated canned food samples that were incubated overnight at 37 or 55 °C confirmed the results of the CFU counts of these samples (cf. Figs. 1 and 2a). After incubation at 37 °C, the read population was dominated by OTU 2 belonging to one of the three initially spiked mesophilic species, the mesophilic spoiler *B. cereus*, whereas after o/n incubation at 55 °C, OTUs of both thermophilic species (*G. stearothermophilus* and *G. thermoglucosidans*) present in the spike, dominated the detected reads (Fig. 2a).

### Detection limit for sequence-based evaluation of bacterial spores

In order to determine the detection limit of bar-coded amplicon sequencing in a model food matrix, the serial dilutions of the spike added to canned food used for determining the CFU counts (Fig. 1) were analyzed by 16S rRNA amplicon sequencing. Normalization of the number of sequence reads was done according to a species present in low amounts in the canned food, in this instance *A. encheleia* (Fig. 2a).

**Fig. 3:**
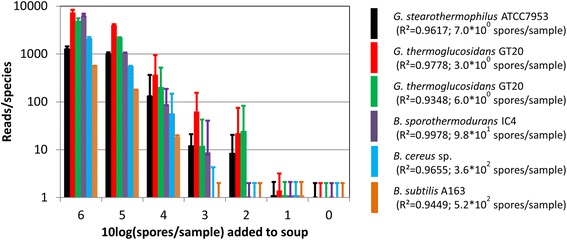
Linear relation between spiked and detected spores and the detection limit (spores/bacterial species/sample giving one read/OTU/sample). Normalization of the “canned food specific” OTU 8 (*Aeromona*s *encheleia*) to 340 reads/sample was applied on all other OTUs. Lines were fitted through points with >5 reads/sample) for 6 spiked OTUs not detected in pure canned food (*R* ^2^ and detection limit indicated behind strain names). Additional file 2 shows normalized (and raw) frequencies of all 2037 OTUs

A reliable linear relationship between the number of added spores and (normalized) reads was observed for the serial dilutions of each of the five spore crops (Table 1, Figs. 2b and 3). In Additional file 3, calculation details of the normalization based on the *A. encheleia* data and the resulting linear regression coefficients (*R*^2^ = 0.945–0.998) and the corresponding detection limits are shown. The calculated detection limit at one sequence read ranges from approximately 3 to 6.3 × 10^2^ spores per 850 μl soup sample for *G. thermoglucosidans* TNO 09.020 and *B. cereus* sp., respectively.

**Table 1:** Correlation and detection limit of mass seq read counts with spore counts

OTUs	Spore spike strain	*y* = *ax* + *b*	Regression coefficient (*R* ^2^)	Det. limit *x* = (*y*−*b*)/*a* (10log(sp/sample) at *y* = 1 read	Spores/sample
OTU 6	*G. stearothermophilus* ATCC7953	*y* = 0.644*x* − 0.5384	0.9617	0.8	7.0E+00
OTU 3	*G. thermoglucosidans* TNO-09.020	*y* = 0.7464*x* − 0.3915	0.9778	0.5	3.0E+00
OTU 5	*G. thermoglucosidans* TNO-09.020	*y* = 0.7222*x* − 0.5543	0.9348	0.8	6.0E+00
OTU 4	*B. sporothermodurans* IC4	*y* = 0.9669*x* − 1.9271	0.9978	2.0	9.8E+01
OTU 2	*B. cereus* TNO-02.0143	*y* = 0.9239*x* − 2.5864	0.9655	2.8	6.3E+02
OTU 7	*B. subtilis* A163	*y* = 1.095*x* − 2.9748	0.9449	2.7	5.2E+02
OTU 1	*B. subtilis* A163 (canned food and spike)	*y* = 0.011*x* + 3.8187	0.0111	−347.2	0.0E+00

The table shows for a tenfold dilution series of spore-mix batches, the linear regression parameters (*a*, *b*, *R* ^2^) for 10log(# spores) and 10log(# reads) plotted on *X*- and *Y*-axis, respectively. *R* ^2^ indicates the quality of linearity while *x* at *y* = 1 yields the detection limit (10log(#spores) for one detected mass sequence read. Calculations are performed for each of the detected known OTUs representing the five different species in the spore-mix. Note that OTU 3 and OTU 5 both represent strain TNO-09.020

Canned food without an inoculum is dominated by sequence reads belonging to OTU 1. When the spore spike is added (a total of 5 × 10^6^ spores), the number of OTU 1 reads is lower (Fig. 2a) indicating a lower relative abundance of the background flora in the spiked canned food in comparison to the non-spiked canned food. The inoculated canned food samples that have been incubated o/n at 37 or 55 °C also show strongly reduced numbers of sequence reads for OTU 1. Due to a similar dilution effect, the number of reads for OTU 8 (*A. encheleia*) is below detectable levels in the samples that have been incubated o/n at 37 or 55 °C.

### Bacterial outgrowth in a ready-to-eat food matrix by amplicon sequencing

**Fig. 4:**
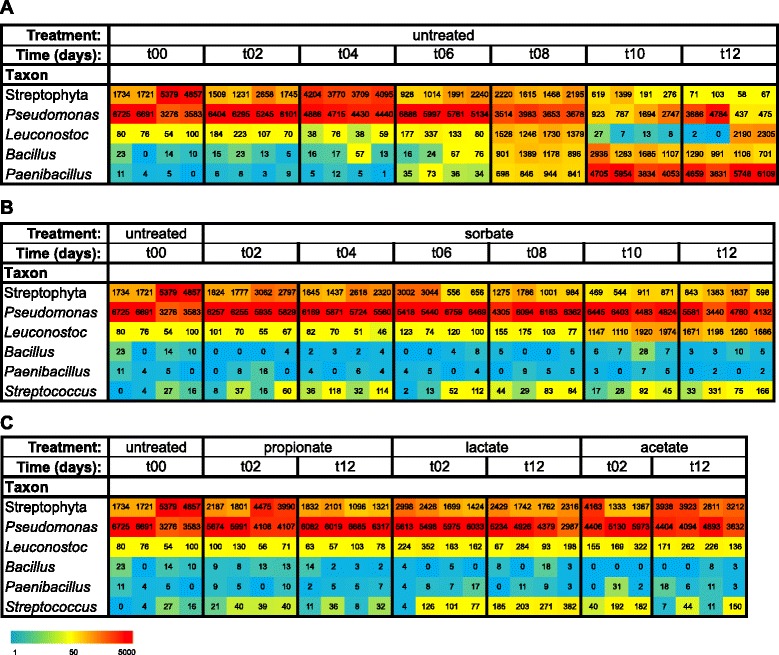
Mass sequence-based analysis of the microbial composition during spoilage of RTE meals. Development of the microbial composition of RTE rice meals was monitored during 12 days (t00–t12) storage/spoilage at 7 °C, by mass sequence-based microbial typing. The *heat map* shows the number of normalized (10^4^ total reads/sample) reads per sample per unique sequence (below the heat map the legend of the color code is provided). For clarity, the actual numbers of normalized reads per sample are also shown. From 422 genera, six were selected showing high abundance and diversity (in time and between treatments) during spoilage time. The panels show microbial composition changes of untreated RTE meal (**a**), RTE meal treated with sorbate (**b**), or RTE meal treated with propionate, lactate, or acetate (**c**). Please note that more genera have been observed than shown in this figure. All observed genera are shown in Additional file 5

The DNA sequences of the 16S rRNA amplicons generated from DNA of untreated and sorbate-treated RTE food samples were obtained by pyrosequencing. Because of the lack of outgrowth of microbes in the propionate-, lactate-, and acetate-treated RTE food samples (Additional file 4), only samples from *t* = 2 and *t* = 12 were analyzed, in order to see whether changes in the population had occurred which were not visible in the CFU counts. Figure 4 shows the number of observed reads linked to the six genera with the highest relative abundance for all sequenced samples. At the start of the experiment, high numbers of reads linked to *Pseudomonas* (33–67 %) and Streptophyta (17–54 %) were observed (Fig. 4a, untreated, t00 days) with the remaining sequences (13–16 %) mainly spread over six to seven bacterial genera (Additional file 5).

In the untreated samples, a shift in the read numbers was observed indicating bacterial activity over the 12-day incubation period at 7 °C. The first genus showing an increase in relative abundance (from t06 until t08) was *Leuconostoc* (Fig. 4a). From t08 on, sequences of the genera *Bacillus* and in particular *Paenibacillus* increased in relative abundance, while *Leuconostoc* read numbers decreased at t10 and partly at t12 (Fig. 4a, t10 and t12). With the relative increase of the abundance of the sequences of the genera *Paenibacillus*, *Bacillus*, and *Leuconostoc*, a general decline in relative abundance of the Streptophyta and *Pseudomonas* sequences is observed (Fig. 4a, t10 and t12). In one sample at t12, however, the number of *Pseudomonas* reads was high, suggesting that in this particular sample, *Pseudomonas* actually did grow.

In contrast to the population dynamics observed in the untreated samples, in the sorbate-treated samples, only one genus clearly increased in relative abundance over time: *Leuconostoc* (Fig. 4b). Numbers of reads belonging to the genera *Bacillus* and *Paenibacillus* did not increase during the incubation, indicating sensitivity of these genera to sorbate, in line with previous observations (see e.g., [15]). In contrast to the untreated and sorbate-treated samples, the propionate-, lactate-, or acetate-treated samples did not show a clear shift in OTU distribution (Fig. 4c), corresponding to the observed lack of outgrowth in these samples (Additional file 4). A minor increase in read numbers was observed for the genus *Streptococcus* in the lactate-treated samples and to an even lesser extent also in the acetate-treated samples.

### Optimization of amplicon sequencing for food matrices with vegetables (chloroplasts)

In order to minimize the influence of chloroplast sequences from vegetables in complex food matrices, a new reverse oligo was designed. The 16S rRNA sequences of common food spoilage bacteria were aligned against a set of various 16S rRNA sequences from chloroplasts from Streptophyta (*Arabidopsis*, *Cucumis*, *Gossypium*, *Hordeum*, *Oryza*, *Solanum*, *Spinacia*, *Triticum*, *Zea mays*). Subsequently, a new reverse oligo was designed (o5958, Additional file 6A), which contained a maximum number of mismatches to the chloroplast sequences, while keeping the highest possible match to common food spoilage bacteria, including members of the phylum Firmicutes (based on RDP Probe Match software (http://rdp.cme.msu.edu/). However, the percentage of identity with most of the other phyla was significantly reduced (Additional file 6B). The forward 16S rRNA amplicon PCR primers used in this study were not modified; they included the forward bar-coded primer(s) o4147-o4382 (Additional file 6A). A clear decrease in the number of Streptophyta sequences was observed in our study with the ready-to-eat food matrix, although a complete exclusion of Streptophyta sequences was not achieved (Additional file 6C).

## Discussion

The aim of this study was to optimize the use of amplicon sequencing as a method for determining spoilage organisms in complex food matrices. Canned food and a RTE meal were chosen as model food matrices as they represent two different relevant food matrices and were suitable for studying different aspects important in food spoilage: the detection limit of amplicon sequencing as detection method for bacterial spore formers in a complex food matrix as well as the possible use of a spikes in order to quantify the detection method (canned food) or the use of amplicon sequencing in following the dynamics of food spoilage at low temperatures caused by spoilage organisms collected during the production process in the presence and absence of commonly used preservatives (RTE rice meal). The results showed that bar-coded amplicon sequencing using the Roche 454 platform is a sensitive and reliable method for detection and simultaneous identification of spoilage organisms, suitable for following population dynamics in time. Moreover, we have described a sensitive normalization procedure utilizing endogenous low-abundant sequences present in the food matrix.

### Normalization of sequence read numbers

An important aspect in quantitative analysis is normalization of the data. In the described RTE rice meal experiment, where relative levels of microbiota members are described, normalization is less important than in the canned food experiment where absolute quantification of microbiota members was desired. In the canned food experiment, we used an OTU sequence found in canned food without inoculum for normalizing all other sequence results. This 16S rRNA sequence belonged to *A. encheleia*, a common water contaminant, which was not detected as CFUs on tryptic soy agar (TSA) and had therefore been inactivated during the canned food production process. This normalization step allowed us to calculate the detection limits for the different spiked bacterial spore species, in terms of the minimum number of spores required to identify a spore contamination. Obviously, a further reduction of the detection limit may be achieved by increasing the sequencing depth (number of amplicon reads per sample).

The normalization procedure and the identified correlation between sequence read numbers and CFUs from the same samples allows for quantification of spores on the basis of amplicon read numbers. A prerequisite for this is a significant number of reads of the OTU used for normalization in all samples. The use of an endogenous OTU can be challenging in heavily contaminated samples as high levels of the contaminant dilute the relative abundance of the OTU used for normalization (as observed in this study for spiked soup samples incubated at 37 and 5 °C). Instead of utilizing an intrinsically present sequence for normalization, it is also possible to spike the samples with a defined amount of unique spores or cells or DNA as an internal control. When such a spike is added, it is important to spike the samples before the DNA extraction step as the DNA extraction step is one of the steps which can introduce sample-to-sample differences which are leveled by normalization. The amount of spike should be adjusted to the expected level of contamination, to allow quantification by amplicon sequencing. The spike is preferably of bacterial origin (to be detectable with the same primers) and unrelated to the expected contamination.

### Comparison between viable count determination and amplicon sequencing results

Comparisons between sequence abundance and colony-forming units should be taken with great care. Differences between the two methods can result from variable DNA extraction efficiencies among bacterial species and spores, the presence of dead cells, and selective outgrowth of species on nutrient agar plates. However, detailed comparison between the two methods yields interesting information about the importance of these factors. A comparison could be made with respect to the influence of the food matrix present in the can. The CFU count of the spiked spores is affected by the canned food matrix by approximately 1 log factor compared to direct culturing of the spore spike (Fig. 1). The DNA extraction efficiency, however, is not influenced by the canned food matrix (Additional file 7A). After normalization of the sequence data, there are hardly any differences between the number of sequence reads from the spike in the presence or absence of canned food (Additional file 7B).

One explanation for the negative effect of the canned food matrix on the CFU count is that spores bind to food particles and are lost in the purification steps, resulting in an underestimation of the CFU count in the canned food matrix. This explanation is however unlikely since the same samples have been used for the CFU determination and the DNA extractions. Another explanation could be that the canned food matrix is preventing efficient germination of the spiked spores. Since the spores were taken out of the canned food matrix and are left to germinate on the TSA plates, this is also an unlikely explanation. The most likely explanation is that multiple spores attached to a food particle are counted as one CFU, resulting in an underestimation of the CFU count in the canned food matrix.

In the RTE rice meal study, there also appeared a correlation between the increase of CFUs in time (Additional file 4) and the rise in the relative number of sequence reads (Fig. 4a), especially for the well-known spoilage species *Leuconostoc*, *Bacillus*, and *Paenibacillus*. For confirmation, eight morphologically distinct colonies were typed by ribosomal 16S rRNA sequencing, and all identified species were also found in the mass sequencing data (Additional file 8). Quantitative comparisons cannot be made, however, as calibration curves based on absolute CFU numbers (on TSA and de Man, Rogosa and Sharpe Agar (MRSA)) and OTU read numbers are not available for each genus.

### Factors influencing the detection limit of 16S rRNA sequencing

The linear correlation between added spores and detected sequence reads after normalization in the canned food experiment (Figs. 2b, 3, and 4b) allowed the calculation of the detection limits for each added species (Fig. 3). The variation in detection limit in the presence of canned food (Figs. 2b and 3) is directly associated with the differences in detection of the species in the absence of canned food (Fig. 2a). Since equal number of spores of the five species were added (determined via spore counting in a Bürker-Türk counting chamber), this reflects differences in the efficiency of detection by sequencing. Such differences can be attributed to differences in DNA extraction efficiency, to variation in rRNA gene copy numbers, or to differences in DNA amplification or to a combination thereof.

In order to distinguish between these possibilities, individual DNA extractions of the five bacterial spore batches were performed, starting with equal numbers of spores (as determined by using a Bürker-Türk counting chamber). The amount of extracted DNA as determined by a universal quantitative PCR [16] indicated large differences in the DNA extraction efficiency with *G. thermoglucosidans* being isolated with more than 3 log units (more than 1000-fold) higher efficiency than *B. cereus* (Additional file 9A).

Amplicon sequence analysis of a mixture of the individually isolated DNAs indicated that the differences in DNA extraction efficiency are also reflected in the number of sequence reads (Additional file 9B). The only exception is *B. subtilis* which is now detected in numbers similar to *B. sporothermodurans* and *G. stearothermophilus* (Additional file 9B), whereas the DNA extraction appeared ~2 log units less efficient (Additional file 9A).

Amplicon sequencing of a mixture from the individually isolated DNAs, in which the amount of DNA for each species was equalized based on the quantitative PCR (qPCR) results (Additional file 9A) indicated that on the basis of quantitative PCR, it is possible to normalize the number of sequence reads for each species (Additional file 7). Only *B. cereus* appears still slightly underrepresented, but the ~2.5 log differences between *G. thermoglucosidans* and *B. cereus* had been reduced to maximally 1 log. Together, these data indicate that differences in DNA extraction efficiency are the most important cause for the different representation of the used bacterial species when mixed in equal numbers. Although spores have been counted under the microscope prior to the experiment, it is possible that *B. cereus* spores are recovered less efficient due to their relatively high hydrophobicity. In addition, variations in the spore maturation, spore core water content, and the number and nature of DNA-binding proteins may result in differences in DNA extraction efficiency. Therefore, it will be very difficult to develop a suitable DNA extraction with a comparable efficiency for all bacterial spores. In order to explain the remaining bias in reads in the samples corrected for the amount of extracted DNA (right panel, Additional file 10), we checked the number of copies of 16S rRNA genes per genome. Based on the available complete genomes of bacilli and geobacilli, the average number of 16S rRNA gene copies varies from 8–14 (Additional file 11). Specific information for *B. sporothermodurans* and *G. stearothermophilus* is unavailable, but information on the genus level indicates that *Bacillus* and *Geobacillus* have a comparable number of 16S rRNA gene copies (Additional file 11). It remains therefore unclear why DNA isolated from *B. cereus* is not only extracted inefficiently but also leads to a relatively inefficient synthesis of amplicons.

### Applicability of 16S rRNA sequencing as method for detection and identification of spoilage organisms

The observed detection limit of 16S rRNA bar-coded amplicon sequencing holds well against other culture-independent (molecular) detection methods such as qPCR [17] with the advantage that in contrast to qPCR, the method has no specific targets but detects all present spoilage bacteria. The current sensitivity of the method is suitable for early detection of spoilage organisms as well as following population dynamics. However, for detection of extremely low-abundant organisms, e.g., pathogens, the method is less suited, as are all (non-specific) detection methods which do not include specific enrichment for the target species.

In order to improve 16S rRNA sequence-based detection of spoilage bacteria, several options are available. The first is to further minimize the influence of chloroplast sequences by optimizing oligo combinations. Another way for improving the sensitivity of 16S rRNA sequence-based detection method is to increase the number of analyzed sequences. By increasing the sequence read numbers, also lower abundant sequences can be detected. The Illumina technology is capable of producing much higher number of sequences than the method used in this study (pyrosequencing) and could therefore be applied to improve the sensitivity [18, 19]. However, a drawback of the Illumina platform is the reduced read length compared to pyrosequencing (the current study yields ~450-bp amplicons, spanning the V5–V7 region, whereas the most commonly used Illumina amplicon spans the shorter V4 region, ~200 bp [20].

### Evidence for multiple OTUs in one bacterial strain

The finding of the seven OTUs from the spore spike containing spores of all five bacterial species indicated a contamination or the presence of multiple 16S rRNA sequences within at least one species. Alignment of the unique sequences represented by the OTUs indicated that two OTUs were matching with *B. subtilis* A163 and two OTUs with *G. thermoglucosidans* TNO-09.020. Evidence for the existence of these two different 16S rRNA sequences was found in the available genome sequences of TNO-09.020 [21] and A163 (Jos Boekhorst, personal communication). Alignment of OTU 1 and OTU 7 with contigs of the genomic sequence of *B. subtilis* A163 revealed identity of the OTUs with one genomic sequence and a sequence obtained from 16 rRNA sequence typing, respectively (Additional file 12A). Similarly, alignment of OTU 5 and OTU 3 with the genome sequence of *G. thermoglucosidans* TNO 09.020 revealed at least two different genomic sequences that corresponded to the sequences of the identified OTUs (Additional file 12B). Consequently, a contamination in the spore spike is very unlikely.

## Conclusions

The current study assessed the deployment of bar-coded amplicon sequencing for the detection of bacterial spoilage organisms within complex food matrices. In this work, methods were developed and optimized for DNA extraction, amplicon synthesis, and data normalization. Clear evidence for the usefulness of bar-coded amplicon sequencing was obtained, based on the detailed typing of members of the spoilage microbiota and low detection limit of the method (on average 2 × 10^2^ ml^−1^). Normalization methods allowing sample-to-sample comparisons were applied on the basis of background DNA from an inactivated bacterial water contaminant. A limitation of the method for accurate quantitative determinations is variation in DNA extraction of spores.

## Methods

### Complex food matrices and spoilage conditions

#### The canned food matrix

Canned creamy mushroom soup (canned food product, purchased in The Netherlands) was used as food matrix for the assessment of bar-coded amplicon sequencing as a feasible method for detection and quantification of bacterial spores. In order to validate the method, a spike of defined bacterial spores was used. Spore crops were purchased for *G. stearothermophilus* ATCC 7953 (Mesa Laboratories, Denver, CO) or produced according to methods described by Zhao et al. [12] for *B. subtilis* A163 [22], *B. sporothermodurans* IC4 [23], *B. cereus* TNO 02.0143 (TNO food product isolate), and *G. thermoglucosidans* TNO-09.020 [21]. Spore suspensions were mixed in equivalent amounts based on spore counts, as determined by a hemocytometer according to methods described by Kort et al. [22]. In summary, six randomly selected squares were counted, with a surface area of 0.0025 mm^2^ and a depth of 0.01 mm each. Spore suspensions containing all five species were added to aliquots of 20 g of creamy mushroom soup in stomacher filter bags (BagFilter S/25, Interscience, France), in tenfold dilutions to final concentrations ranging from 1 × 10^6^ to 1 × 10^1^ spores of each species per ml canned food. After mixing the spores with the canned food, a Pulsifier treatment was performed (1 min in a Pulsifier, LED Techno, Den Bosch, The Netherlands) in order to release the spores or bacteria from the canned food matrix. After passing through the filter of the stomacher bag, the samples were used for determination of CFU counts on TSA plates incubated at 37 or 55 °C, and three samples of each mixture (850 μl, corresponding to 8.5 × 10^5^ to 8.5 × 10^0^ spores each) were taken for total DNA extraction. The outgrowth of added bacterial spores was studied at 37 or 55 °C. Four aliquots (20 g each) of creamy mushroom soup were spiked with the spore mixture until a final concentration of 1 × 10^6^ spores per ml. Two spiked aliquots were incubated o/n at 37 °C and the other two spiked aliquots were incubated o/n at 55 °C (Additional file 13), a starting number of 8.5 × 10^5^ spores of each bacterium) for total DNA extraction.

#### The ready-to-eat food matrix

The bacterial outgrowth was studied in a ready-to-eat (RTE) meal consisting of fried rice, vegetables, and meat. This meal was purchased in a local supermarket and was packaged in a disposable plastic container, sealed airtight with a plastic cover. The meal consisted of rice (rice, water, 62 %), leek (12 %), pork meat (7 %), ham (pork meat, salt, potato starch, soy protein, aroma, 7 %), egg (7 %), vegetable oil, yeast extracts, salt, and sugar. Several packages of the ready-to-eat rice meal of the same batch were mixed and divided over five portions, with one serving as a control, and the others treated with different organic acids, including 0.3 % propionic acid >99 % (Acros Organics, Belgium), 0.1 % potassium sorbate (Acros Organics, Belgium), 2.5 % acetic acid glacial >99 % (Fisher Scientific, United Kingdom), or 2.5 % Purac FCC 80 (lactic acid; Corbion, The Netherlands). The pH of all five batches was adjusted to 5.5 with HCl, and the batches were then distributed into samples of 15–20 g for each sampling point (see Additional file 14). Each sample was placed into a plastic stomacher filter bag (BagFilter S/25, Interscience, France). The plastic stomacher bags were sealed with a clip and stored at 7 °C until sampling. Every 2 days, two samples were taken from the 7 °C incubator, and three volumes of peptone-physiological salt solution (0.85 % (*w*/*v*) NaCl, 0.1 % peptone in demineralized water) were added to each portion, followed by treatment with a stomacher (IUL Masticator, LA-Biosystems, Waalwijk, The Netherlands) for 60 s onto TSA (Oxoid CM0131, 2 days, 37 °C for isolation of all aerobic bacteria) and MRSA (Oxoid CM0361, 2 days, 37 °C under microaerophilic conditions for isolation of lactic acid bacteria) plates.

Based on the observed CFU counts, samples were selected for 16S rRNA gene sequence analysis in order to review the microbial population at that stage. All samples of the untreated and sorbate-treated samples were selected for sequence analysis in duplicate (i.e., from samples 1–2, 3–4, 13–14, 23–24, 33–34, 43–44, 53–54 and 7–8, 17–18,27-28, 37–38, 47–48, 57–58, indicated in bold in Additional file 14, two 300 μl aliquots were analyzed individually). From the propionate-, acetate-, and lactate-treated samples, only time points *t* = 2 (samples 5–6, 9–10, 11–12) and *t* = 12 (samples 55–56, 59–60, 61–61) were analyzed in duplicate.

#### Sample pre-treatment and DNA extraction

Prior to the total DNA extraction, spores were isolated from the canned food samples by adding 150 μl of α-amylase (Sigma Aldrich A7595-250ML 240 l) to each 850-μl sample and subsequent incubation for 6 min at 65 °C, followed by addition of 20 μl proteinase K (15 mg/ml, Sigma P2308). After 2 min of centrifugation in an Eppendorf microcentrifuge, the supernatant was discarded and the pellets were frozen at −20 °C for subsequent DNA extractions. DNA extractions were performed using phenol bead beating in combination with the Agowa Mag mini DNA extraction kit (catalogue 40401, LGC genomics, Berlin, Germany).

To the frozen canned food pellets containing the bacterial spores, a volume of 500 μl phenol, 600 μl zirconium beads, and 500 μl Agowa lysis buffer was added. To each 300-μl RTE meal sample, 600 μl zirconium beads (diameter 0.1 mm, catalogue 11079101z, Biospec Products, Bartlesville, OK), 400 μl lysis buffer (Agowa Mag mini DNA extraction kit), and 300 μl phenol, pH 8.0 (Phenol solution BioUltra, catalogue P4557, Sigma Aldrich, St Louis, MO), were added.

Mechanical disruption of bacterial cells or spores was done by bead beating for 2 min in a mini-beadbeater-8 cell disruptor (Merlin Bio-products, Breda, The Netherlands) at setting fast (homogenize). After bead beating, the samples were cooled on ice prior to a 10 min 10,000 RPM (9300 RCF) centrifugation step. After another phenol extraction step of the aqueous phase, 500 μl of the aqueous phase (corresponding to 0.7 or 1 original volume of the ready-to-eat and canned food samples, respectively) was transferred to a new centrifugation tube prefilled with 1000 μl binding buffer (Agowa) and 20 μl magnetic beads (Agowa). After mixture, the suspension was left for 10 min to allow binding of the chromosomal DNA to the magnetic beads. After washing the beads according to the Agowa Mag mini DNA extraction protocol, the DNA was extracted from the beads with 63 μl elution buffer (Agowa) according to the manufacturer’s instructions. For the canned food samples, this corresponds to DNA from 8.5 × 10^5^ spores.

#### Bar-coded 16S amplicon sequencing

Quantitative 16S-PCR was performed to determine the relative amount of bacterial template in the isolated DNA samples [16]. Based on this quantification, a maximum of 5 μl of template was used for the generation of a 16S rRNA gene amplicon library spanning variable regions V5–V7 [24]. Sequence analysis of the amplicon library was performed on a 454 GS-FLX-Titanium Sequencer (Life Sciences (Roche), Branford, CT).

#### Sequence processing and analysis

FASTA-formatted sequences and corresponding quality scores were extracted from the .sff data file generated by the GS-FLX Titanium sequencer using the GS Amplicon software package (Roche, Branford, CT) and processed using modules implemented in the Mothur v. 1.22.2 software platform [25]. Samples yielding insufficient reads (<1000) were excluded in further analysis. The sequences were de-noised using a pseudo-single linkage algorithm with the goal of removing sequences that are likely due to pyrosequencing errors (“pre.cluster” command) [26]. Potentially chimeric sequences were detected and removed using the “chimera.uchime” command [27]. High-quality aligned sequences were classified using the RDP-II naive Bayesian classifier [28]. Aligned 16S rRNA gene sequences were clustered into operational taxonomic units (OTUs) using the average linkage clustering method.

For the study on the ready-to-eat meal, OTUs were defined by 97 % identity (“3 % OTU’s”), and each OTU was classified at tax level 6, resulting in sequence read frequencies for 422 different genera. For the study on the canned food, OTUs were defined by 100 % identity (facilitating comparison of unique sequence reads with the known 16S sequences of the five applied spore isolates), resulting in read frequencies for 2037 OTUs. For both studies the total number of sequence reads per sample was normalized to 10,000 resulting in normalized read frequencies per genus (ready-to-eat meal study) or per OTU (canned food study).

For determination of the detection limit in the canned food experiment, the normalization was performed by using the average number of *A. encheleia* sequences in the unspiked canned food samples as reference and adjusting all other frequencies of *A. encheleia* in the spiked samples to this average (assuming a constant distribution of inactivated *A. encheleia* cells in the single can of canned food that was used, calculations in Additional file 3).

#### Calculation of detection limits amplicon sequencing in a canned food matrix

Using the above described normalized data for (non-)spiked canned food data (normalized reads/OTU/sample with total reads/sample = 10,000, followed by a second normalization based on *A. encheleia* reads in (non-)spiked samples), we plotted 10log(reads/OTU/sample) against 10log(spores/sample) for each of the five pairs of OTU/spore-crop and calculated the detection limit (spores/sample) at one read/OTU using linear regression. Samples yielding no reads (at higher spore dilutions) were excluded from the linear regression (Additional file 3). Note that detection limits linearly increase at higher read/OTU levels (e.g., tenfold higher at ten instead of one read/OTU).

### Availability of supporting data

The sequence data are available in the European Nucleotide Archive (ENA) under accession number PRJEB7698 (http://www.ebi.ac.uk/ena/data/view/PRJEB7698).

## Additional files

Additional file 1:
**Processing of sequences and OTUs obtained in this study. A.** Overview of the number of samples and sequences for both food matrices. **B.** Overview of the sequence processing of the canned food samples. **C.** Overview of the sequence processing for the RTE rice meal samples. The number of unique sequences of the RTE rice meal samples has been normalized to 10,000 (arbitrarily chosen). (XLSX 12 kb) Additional file 2:
**Normalized data of mass sequence-based microbial typing and semi-quantitative detection on a spore spoilage model for a canned food.** The file is an Excel file containing the normalized (and raw-total) frequencies of all OTUs for all samples in Fig. 3. (XLSX 421 kb) Additional file 3:
**Calculation of regression data as summarized in Table** **1** **.** The Excel file contains for tenfold dilution series of spore-mix batches, the calculations for obtaining the linear regression parameters (a, b, R2) for 10log(# spores) and 10log(# reads) plotted on *X*- and *Y*-axis, respectively. R2 indicates the quality of linearity while *x* at *y* = 1 yields the detection limit (10log(#spores) for one detected mass sequence read. Calculations are performed for each of the detected known OTUs representing the five different species in the spore-mix. Note that OTU 3 and OTU 5 both represent strain TNO-09.020. (XLSX 929 kb) Additional file 4:
**Viable counts during spoilage of RTE rice meals in the absence/presence of food preservatives.** Colony-forming unit (CFU) counts of the various RTE meal samples during 12 days storage/spoilage at 7 °C. The upper and lower panels show CFU counts on TSA and MRSA plates, respectively. The five different sample treatments are indicated from left to right: untreated (only pH adjusted), sorbate, propionate, lactate, and acetate, respectively. Note that each CFU count (each bar) is derived from a unique sample. This explains why there are differences between samples taken at the same time (e.g., B, t08 where one untreated and one sorbate-treated sample showed clear growth, but the other untreated and sorbate-treated sample did not show growth). The dashed black lines depict the detection limit: bars below this dashed line represent samples where no growth was observed. (PPTX 424 kb) Additional file 5:
**Normalized data of mass sequence-based analysis of the microbial composition during spoilage of RTE meals.** The figure is an Excel file containing the normalized (and raw-total) frequencies of all OTUs for all samples in Fig. 4. (XLSX 208 kb) Additional file 6:
**Primer composition and specificity. A.** Sequence composition of the primers used for 16S rRNA amplicon generation. Each forward primer (o4147 till o4382) contains a unique barcode tag of ten nucleotides, indicated as nnnnnnnnnn. The commonly used reverse primer o4146 and the newly designed reverse primer o5958 for exclusion of chloroplast sequences are also shown. The reverse primers do not carry a barcode. *Roche-TitanA/B-sequence is defined as: 26 bp “seq.primer,” 4 bp “key,” 151 “barcodes” (10 bp). **B.** Calculated specificity of the used primers indicated as fraction of RDP 16S sequences recognized (perfect match with published 16S rRNA sequences) by the used primers. **C.** Experimental evaluation of the reduction of the number of Streptophyta amplicon sequence reads by alternative 16S rRNA amplicon primer design (o5958). The newly designed “non-Streptophyta” 16S rRNA reverse oligo (o5958) was compared with a “universal” 16S rRNA reverse oligo (o4146, gray) during generation of the 16S amplicon on replicate DNA samples isolated from two time points of untreated ready-to-eat rice meal (4 and 8 days). Percentages of sequence reads obtained for the genera *Pseudomonas*, Streptophyta (chloroplasts), and rest (all other genera) are shown. A clear decrease in the percentage of Streptophyta reads is observed with the use of o5958. (PPTX 462 kb) Additional file 7:
**Influence of canned food matrix on DNA extraction and sequence read numbers.** **A.** Comparison of the efficiency of DNA extraction from the spike mixture (10^6^ spores of each species), the spore mixture (10^6^ spores of each species) added to canned food, and from the canned food control. DNA extraction efficiency was determined by universal 16S rRNA qPCR analysis [18]. **B.** Influence of the canned food matrix on the sequence reads of the spike mixture. No clear differences between sequence reads from the spike mixture isolated from a physiological salt solution and isolated from the canned food matrix were observed (left and middle panel, respectively). No sequences corresponding to the spike mixture, other than OTU 1, were observed in the unspiked canned food matrix (right panel). (PPTX 440 kb) Additional file 8:
**Classification by 16S rRNA typing of various colony types found during spoilage of RTE meals.** Morphologically, distinct colony types were subjected to ribosomal 16S sequencing (Baseclear, Leiden, The Netherlands) and compared to the 16S ribosomal database (RDP, Seqmatch tool). The columns indicate sample Nr and description (see Table 1), 16S Seq identity (1.000 = perfect RDP Seqmatch) and top match (RDP Seqmatch species with highest Seq identity), and abundance (an indication of the quantity of colonies found over the complete CFU screening of Fig. 1: high = commonly found, low = found incidentally, unique = found once). (PPTX 502 kb) Additional file 9:
**DNA extraction efficiency of five bacterial spore crops measured by universal 16S rRNA qPCR and 454 mass sequencing. A.** Individual DNA extraction efficiency of five different but equally dense (as determined by spore counting in a Bürker-Türk counting chamber) bacterial spore suspensions measured by universal 16S rRNA qPCR [16]. **B.** DNA isolated from individual spore crops was mixed in equal volumes and subjected to mass sequence analysis. The number of reads per OTU is given, after normalization to a total of 10,000 reads in the sample. (PPTX 436 kb) Additional file 10:
**Relative read frequency in (normalised) spore (DNA)-mixes of five species.** A comparison between the number of sequence reads from a mixture of the five bacterial spores **(A)** and DNA extracted from an equal amount of individually extracted spore crops **(B)** was performed. In **(C)**, the number of sequence reads is equalized by adjusting the amount of spore DNA on the basis of the extraction efficiency (Additional file 9A). (PPTX 514 kb) Additional file 11:
**Copy number of various** ***Bacillus*** **genera.** Using our *Geobacillus thermoglucosidans* TNO 09.020 16S rRNA V5-V7-sequence (OTU 3) as a BLASTN query, we reported the 16S rRNA copy numbers in various sections of the Genbank full genome library. **A)** For the *Bacillus* genera within the *Firmicutes* class, the number of genera with genome sequence available), the total number of 16S rRNA copies within those strains, and the average number of 16S copies are shown. **B)** The number of 16S rRNA copies within complete genomes of *B. cereus*, *B. subtilis*, and *Geobacillus* sp. is indicated. **C)** For *Geobacillus* sp., an overview of complete and draft genomes is shown. A clear underrepresentation of the number of 16S rRNA copies within the draft genomes is visible. (PPTX 550 kb) Additional file 12:
**Sequence alignments of A163 and TNO-09.020 16S rRNA gene sequences indicating the presence of different 16S rRNA gene copies within the genomes of these species.** **A)** Sequence alignment of OTU 1 and OTU 7 with the corresponding sequences of the genomic assembly fragment of *B. subtilis* A163 (kindly provided by Jos Boekhorst, NIZO, Ede, The Netherlands) and the sequence obtained via a 16S rRNA gene sequence typing (“PCR fragment,” Baseclear, Leiden, The Netherlands). **B)** Sequence alignment of OTU 3 and OTU 5 with the corresponding sequences of the genome sequence of *G. thermoglucosidans* TNO 09.020. (PPTX 3612 kb) Additional file 13:
**Detailed sampling scheme for a spore spoilage model for canned food.** The scheme summarizes batches of canned food (creamy mushroom soup), serial dilutions of a spore mixture (of five known *Bacillus* species), serial dilutions of the spore mixture in canned food, and spore/canned food mixtures incubated at 37 or 55 °C. CFU counts were determined for each individual sample, whereas chromosomal DNA for 16S rRNA bar-coded amplicon sequencing was isolated from two or three aliquots (indicated in the column “# replicates”). (PPTX 383 kb) Additional file 14:
**Sampling scheme for spoilage of RTE meals in the absence/presence of food preservatives.** Each sample is indicated by a number. CFU counts were determined for each individual sample, whereas chromosomal DNA for 16S rRNA bar-coded amplicon sequencing was isolated from two aliquots of selected individual samples (indicated in bold) to serve as biological duplicates. Time is shown as number of days inoculation at 7 °C. (PPTX 335 kb)

## Abbreviations

CFU: colony-forming unit
MRSA: de Man, Rogosa and Sharpe Agar
OTU: operational taxonomic unit
qPCR: quantitative PCR
RPD: ribosomal database project
RTE: ready-to-eat
SD: standard deviation
TSA: tryptic soy agar
