# Supplementary material for: Amplicon sequencing for the quantification of spoilage microbiota in complex foods including bacterial spores
Source: Microbiome. 2015 Jul 27;3:30. doi: 10.1186/s40168-015-0096-3 (PMC4515881; doi:10.1186/s40168-015-0096-3)
Supplement: Additional file 4: — Viable counts during spoilage of RTE rice meals in the absence/presence of food preservatives. Colony-forming unit (CFU) counts of the various RTE meal samples during 12 days storage/spoilage at 7 °C. The upper and lower panels show CFU counts on TSA and MRSA plates, respectively. The five different sample treatments are indicated from left to right: untreated (only pH adjusted), sorbate, propionate, lactate, and acetate, respectively. Note that each CFU count (each bar) is derived from a unique sample. This explains why there are differences between samples taken at the same time (e.g., B, t08 where one untreated and one sorbate-treated sample showed clear growth, but the other untreated and sorbate-treated sample did not show growth). The dashed black lines depict the detection limit: bars below this dashed line represent samples where no growth was observed. (PPTX 424 kb) [file 40168_2015_96_MOESM4_ESM.pptx]

## Slide 1
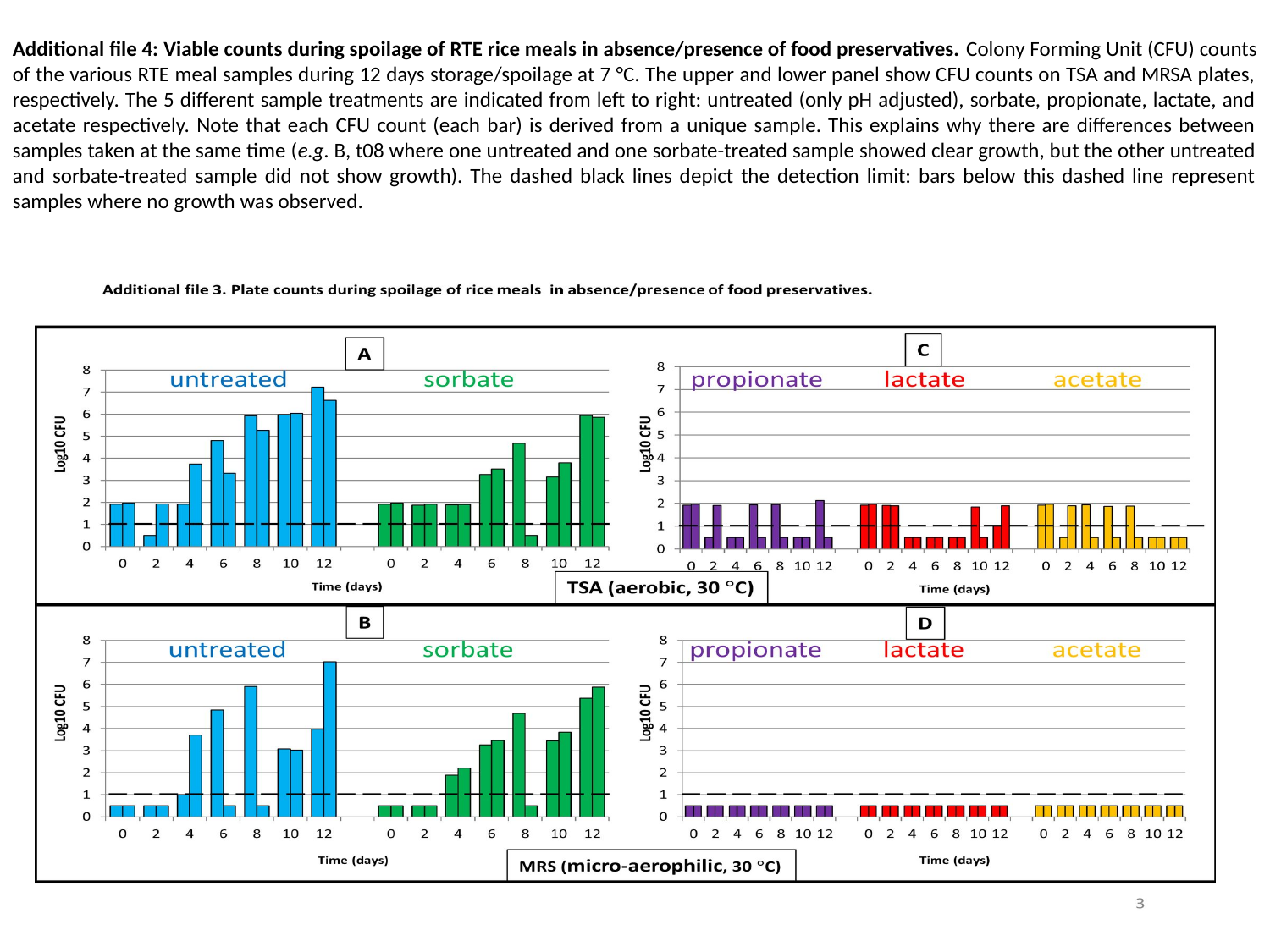

Additional file 4: Viable counts during spoilage of RTE rice meals in absence/presence of food preservatives. Colony Forming Unit (CFU) counts of the various RTE meal samples during 12 days storage/spoilage at 7 °C. The upper and lower panel show CFU counts on TSA and MRSA plates, respectively. The 5 different sample treatments are indicated from left to right: untreated (only pH adjusted), sorbate, propionate, lactate, and acetate respectively. Note that each CFU count (each bar) is derived from a unique sample. This explains why there are differences between samples taken at the same time (e.g. B, t08 where one untreated and one sorbate-treated sample showed clear growth, but the other untreated and sorbate-treated sample did not show growth). The dashed black lines depict the detection limit: bars below this dashed line represent samples where no growth was observed.
