# Supplementary material for: Amplicon sequencing for the quantification of spoilage microbiota in complex foods including bacterial spores
Source: Microbiome. 2015 Jul 27;3:30. doi: 10.1186/s40168-015-0096-3 (PMC4515881; doi:10.1186/s40168-015-0096-3)
Supplement: Additional file 6: — Primer composition and specificity. A. Sequence composition of the primers used for 16S rRNA amplicon generation. Each forward primer (o4147 till o4382) contains a unique barcode tag of ten nucleotides, indicated as nnnnnnnnnn. The commonly used reverse primer o4146 and the newly designed reverse primer o5958 for exclusion of chloroplast sequences are also shown. The reverse primers do not carry a barcode. *Roche-TitanA/B-sequence is defined as: 26 bp “seq.primer,” 4 bp “key,” 151 “barcodes” (10 bp). B. Calculated specificity of the used primers indicated as fraction of RDP 16S sequences recognized (perfect match with published 16S rRNA sequences) by the used primers. C. Experimental evaluation of the reduction of the number of Streptophyta amplicon sequence reads by alternative 16S rRNA amplicon primer design (o5958). The newly designed “non-Streptophyta” 16S rRNA reverse oligo (o5958) was compared with a “universal” 16S rRNA reverse oligo (o4146, gray) during generation of the 16S amplicon on replicate DNA samples isolated from two time points of untreated ready-to-eat rice meal (4 and 8 days). Percentages of sequence reads obtained for the genera Pseudomonas, Streptophyta (chloroplasts), and rest (all other genera) are shown. A clear decrease in the percentage of Streptophyta reads is observed with the use of o5958. (PPTX 462 kb) [file 40168_2015_96_MOESM6_ESM.pptx]

## Slide 1
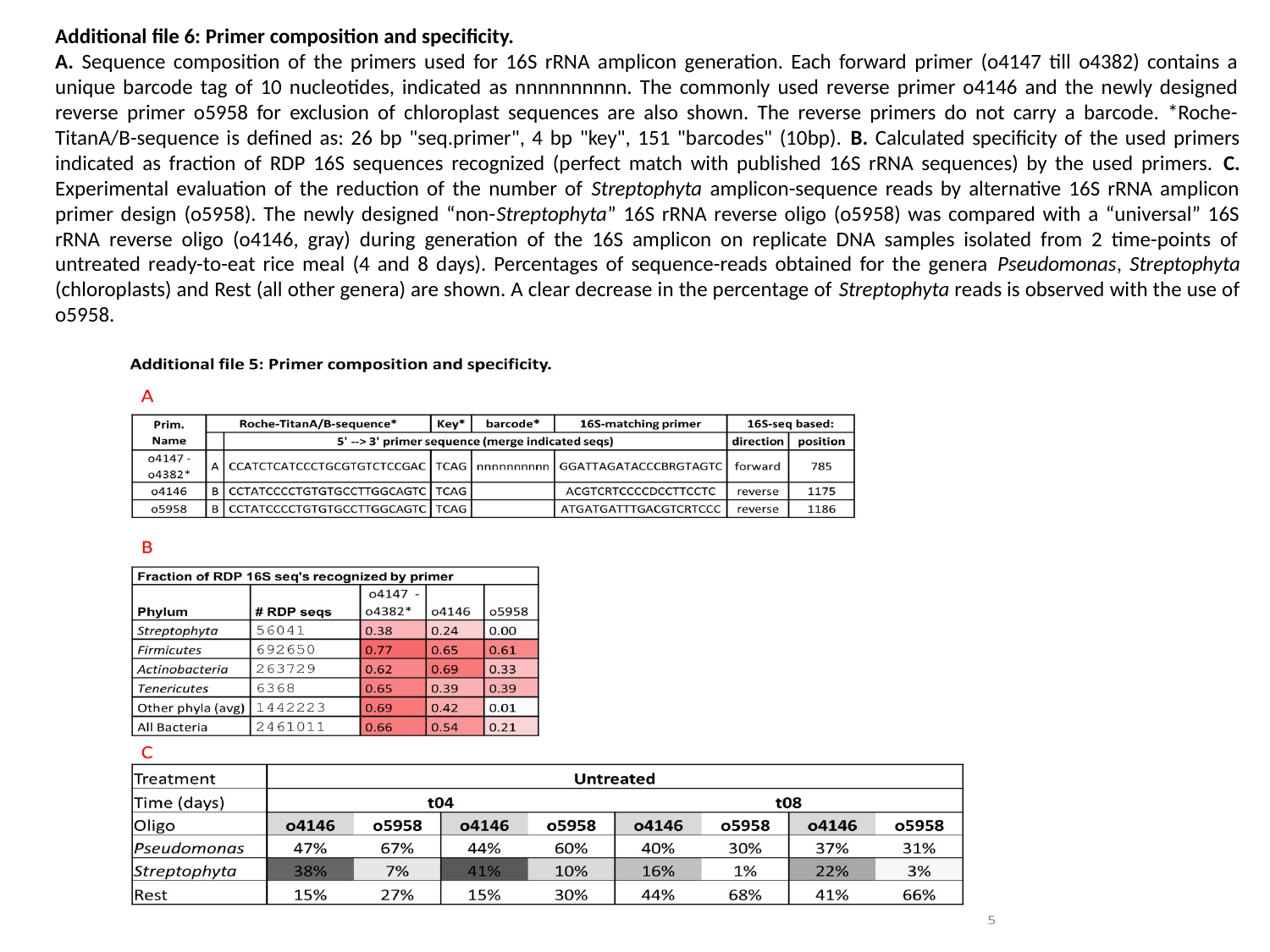

Additional file 6: Primer composition and specificity.
A. Sequence composition of the primers used for 16S rRNA amplicon generation. Each forward primer (o4147 till o4382) contains a unique barcode tag of 10 nucleotides, indicated as nnnnnnnnnn. The commonly used reverse primer o4146 and the newly designed reverse primer o5958 for exclusion of chloroplast sequences are also shown. The reverse primers do not carry a barcode. *Roche-TitanA/B-sequence is defined as: 26 bp "seq.primer", 4 bp "key", 151 "barcodes" (10bp). B. Calculated specificity of the used primers indicated as fraction of RDP 16S sequences recognized (perfect match with published 16S rRNA sequences) by the used primers. C. Experimental evaluation of the reduction of the number of Streptophyta amplicon-sequence reads by alternative 16S rRNA amplicon primer design (o5958). The newly designed “non-Streptophyta” 16S rRNA reverse oligo (o5958) was compared with a “universal” 16S rRNA reverse oligo (o4146, gray) during generation of the 16S amplicon on replicate DNA samples isolated from 2 time-points of untreated ready-to-eat rice meal (4 and 8 days). Percentages of sequence-reads obtained for the genera Pseudomonas, Streptophyta (chloroplasts) and Rest (all other genera) are shown. A clear decrease in the percentage of Streptophyta reads is observed with the use of o5958.
