# Supplementary material for: Amplicon sequencing for the quantification of spoilage microbiota in complex foods including bacterial spores
Source: Microbiome. 2015 Jul 27;3:30. doi: 10.1186/s40168-015-0096-3 (PMC4515881; doi:10.1186/s40168-015-0096-3)
Supplement: Additional file 7: — Influence of canned food matrix on DNA extraction and sequence read numbers. A. Comparison of the efficiency of DNA extraction from the spike mixture (106 spores of each species), the spore mixture (106 spores of each species) added to canned food, and from the canned food control. DNA extraction efficiency was determined by universal 16S rRNA qPCR analysis [18]. B. Influence of the canned food matrix on the sequence reads of the spike mixture. No clear differences between sequence reads from the spike mixture isolated from a physiological salt solution and isolated from the canned food matrix were observed (left and middle panel, respectively). No sequences corresponding to the spike mixture, other than OTU 1, were observed in the unspiked canned food matrix (right panel). (PPTX 440 kb) [file 40168_2015_96_MOESM7_ESM.pptx]

## Slide 1
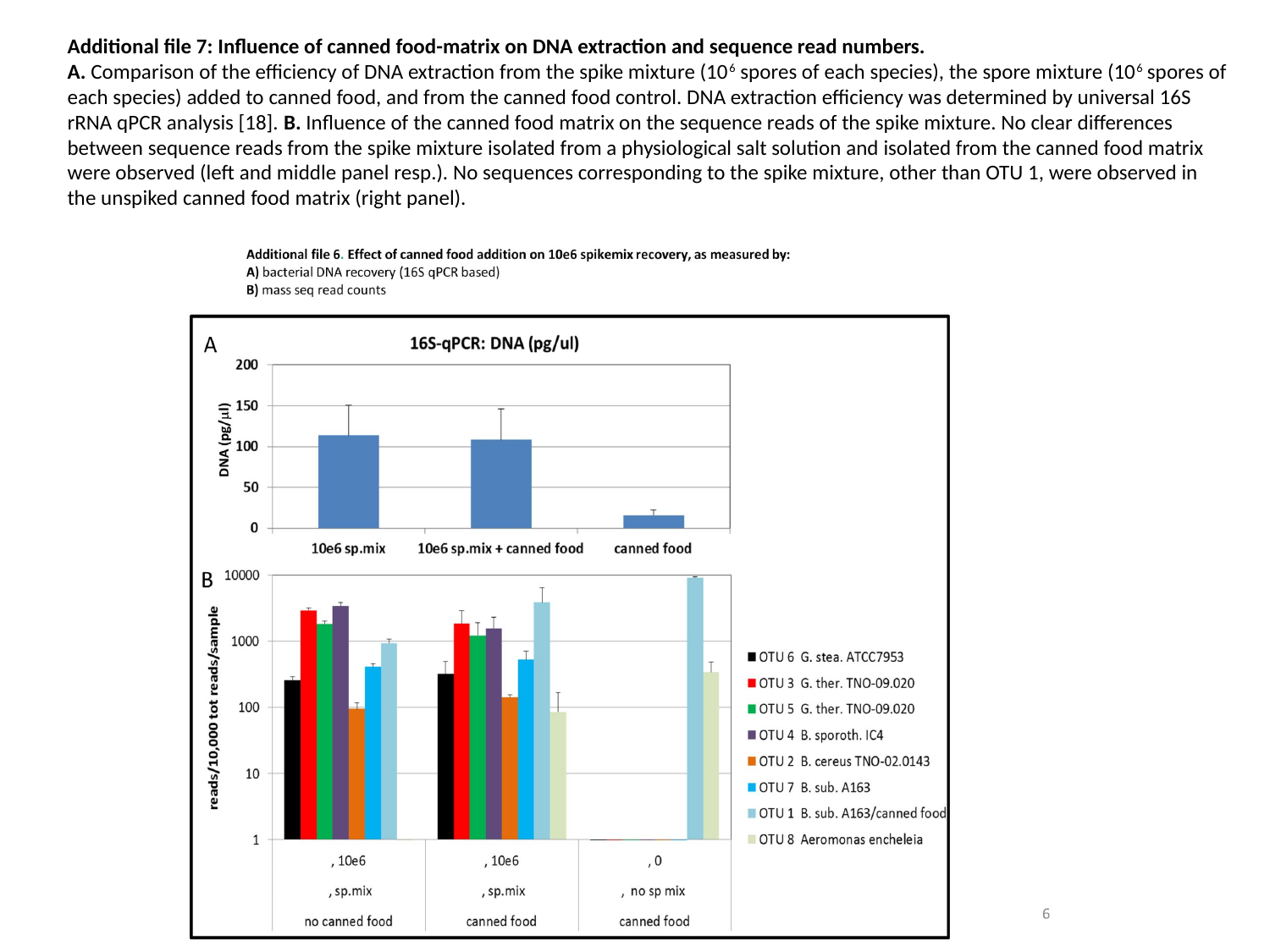

Additional file 7: Influence of canned food-matrix on DNA extraction and sequence read numbers.
A. Comparison of the efficiency of DNA extraction from the spike mixture (106 spores of each species), the spore mixture (106 spores of each species) added to canned food, and from the canned food control. DNA extraction efficiency was determined by universal 16S rRNA qPCR analysis [18]. B. Influence of the canned food matrix on the sequence reads of the spike mixture. No clear differences between sequence reads from the spike mixture isolated from a physiological salt solution and isolated from the canned food matrix were observed (left and middle panel resp.). No sequences corresponding to the spike mixture, other than OTU 1, were observed in the unspiked canned food matrix (right panel).
