# Supplementary material for: Amplicon sequencing for the quantification of spoilage microbiota in complex foods including bacterial spores
Source: Microbiome. 2015 Jul 27;3:30. doi: 10.1186/s40168-015-0096-3 (PMC4515881; doi:10.1186/s40168-015-0096-3)
Supplement: Additional file 8: — Classification by 16S rRNA typing of various colony types found during spoilage of RTE meals. Morphologically, distinct colony types were subjected to ribosomal 16S sequencing (Baseclear, Leiden, The Netherlands) and compared to the 16S ribosomal database (RDP, Seqmatch tool). The columns indicate sample Nr and description (see Table 1), 16S Seq identity (1.000 = perfect RDP Seqmatch) and top match (RDP Seqmatch species with highest Seq identity), and abundance (an indication of the quantity of colonies found over the complete CFU screening of Fig. 1: high = commonly found, low = found incidentally, unique = found once). (PPTX 502 kb) [file 40168_2015_96_MOESM8_ESM.pptx]

## Slide 1
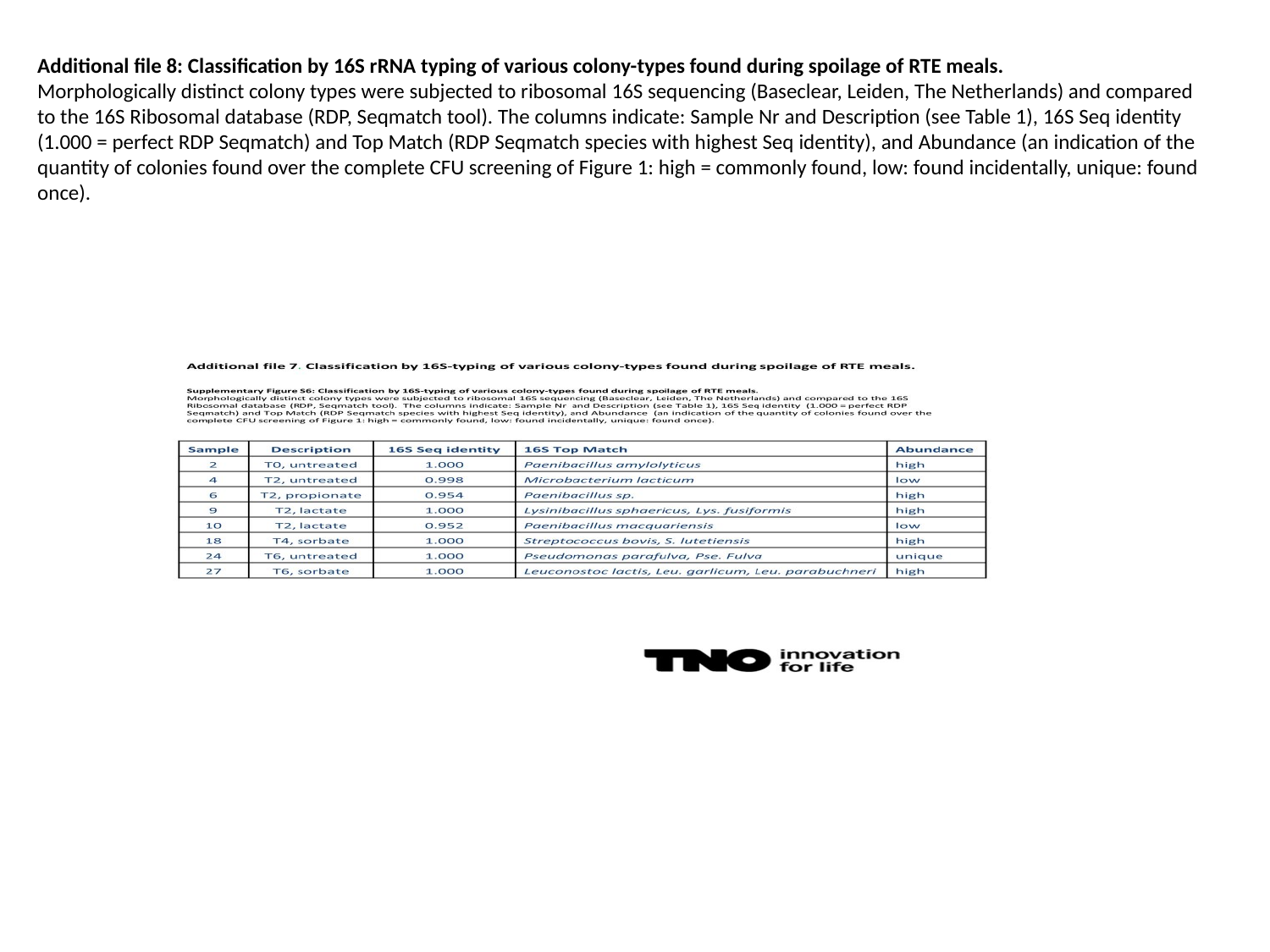

Additional file 8: Classification by 16S rRNA typing of various colony-types found during spoilage of RTE meals.
Morphologically distinct colony types were subjected to ribosomal 16S sequencing (Baseclear, Leiden, The Netherlands) and compared to the 16S Ribosomal database (RDP, Seqmatch tool). The columns indicate: Sample Nr and Description (see Table 1), 16S Seq identity (1.000 = perfect RDP Seqmatch) and Top Match (RDP Seqmatch species with highest Seq identity), and Abundance (an indication of the quantity of colonies found over the complete CFU screening of Figure 1: high = commonly found, low: found incidentally, unique: found once).
