# Supplementary material for: Amplicon sequencing for the quantification of spoilage microbiota in complex foods including bacterial spores
Source: Microbiome. 2015 Jul 27;3:30. doi: 10.1186/s40168-015-0096-3 (PMC4515881; doi:10.1186/s40168-015-0096-3)
Supplement: Additional file 9: — DNA extraction efficiency of five bacterial spore crops measured by universal 16S rRNA qPCR and 454 mass sequencing. A. Individual DNA extraction efficiency of five different but equally dense (as determined by spore counting in a Bürker-Türk counting chamber) bacterial spore suspensions measured by universal 16S rRNA qPCR [16]. B. DNA isolated from individual spore crops was mixed in equal volumes and subjected to mass sequence analysis. The number of reads per OTU is given, after normalization to a total of 10,000 reads in the sample. (PPTX 436 kb) [file 40168_2015_96_MOESM9_ESM.pptx]

## Slide 1
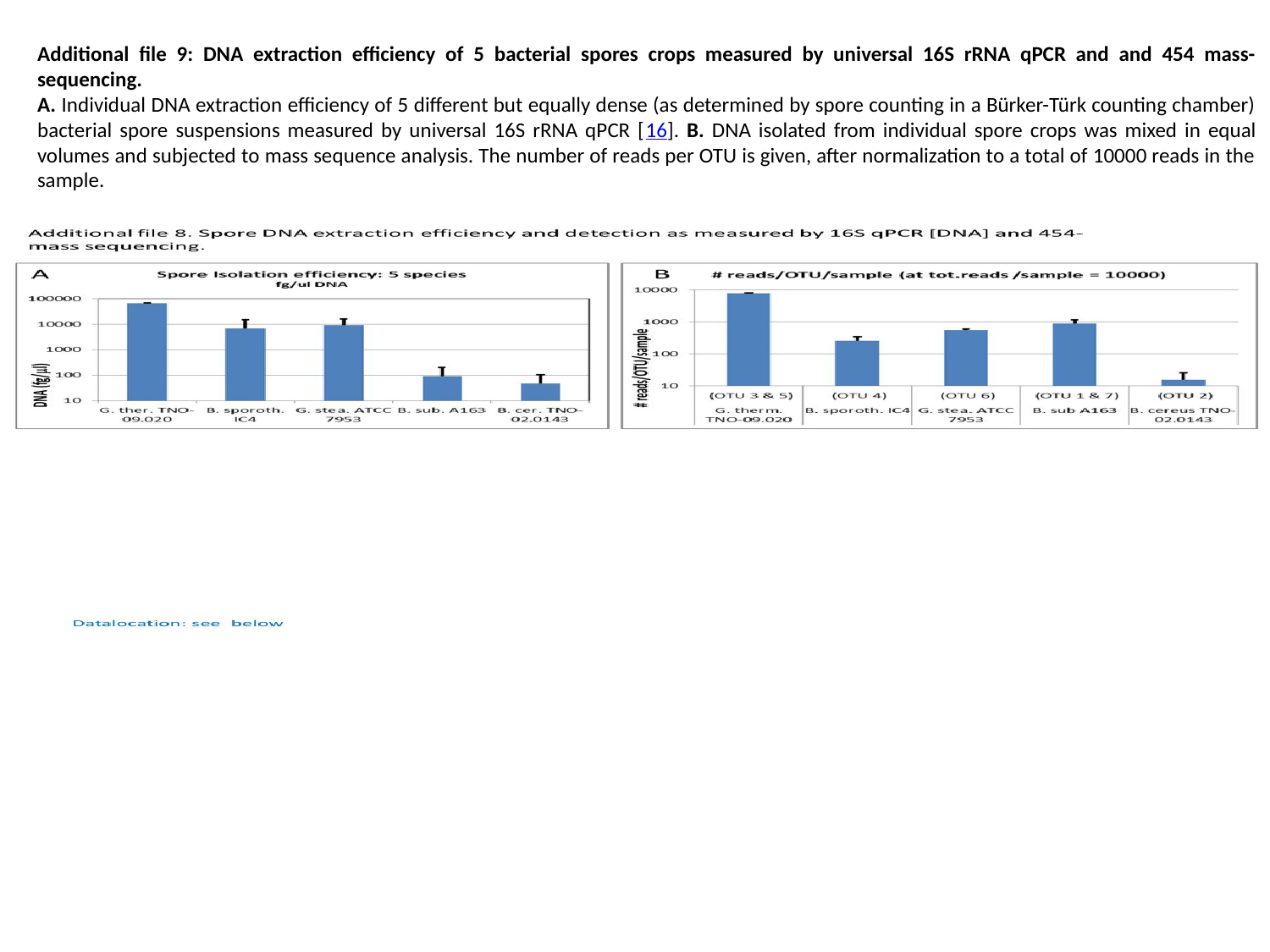

Additional file 9: DNA extraction efficiency of 5 bacterial spores crops measured by universal 16S rRNA qPCR and and 454 mass-sequencing.
A. Individual DNA extraction efficiency of 5 different but equally dense (as determined by spore counting in a Bürker-Türk counting chamber) bacterial spore suspensions measured by universal 16S rRNA qPCR [16]. B. DNA isolated from individual spore crops was mixed in equal volumes and subjected to mass sequence analysis. The number of reads per OTU is given, after normalization to a total of 10000 reads in the sample.
