# Supplementary material for: Amplicon sequencing for the quantification of spoilage microbiota in complex foods including bacterial spores
Source: Microbiome. 2015 Jul 27;3:30. doi: 10.1186/s40168-015-0096-3 (PMC4515881; doi:10.1186/s40168-015-0096-3)
Supplement: Additional file 10: — Relative read frequency in (normalised) spore (DNA)-mixes of five species. A comparison between the number of sequence reads from a mixture of the five bacterial spores (A) and DNA extracted from an equal amount of individually extracted spore crops (B) was performed. In (C), the number of sequence reads is equalized by adjusting the amount of spore DNA on the basis of the extraction efficiency (Additional file 9A). (PPTX 514 kb) [file 40168_2015_96_MOESM10_ESM.pptx]

## Slide 1
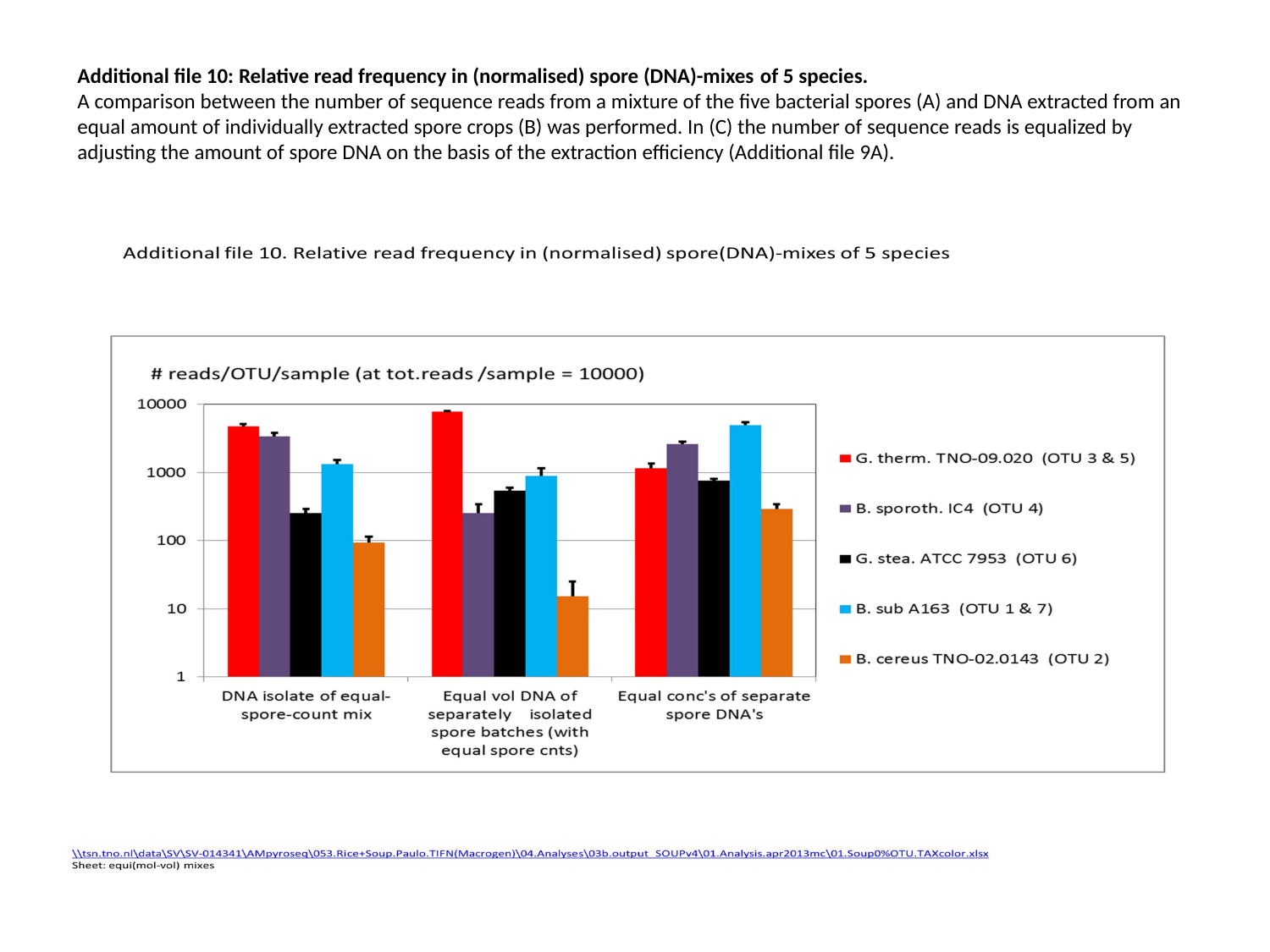

Additional file 10: Relative read frequency in (normalised) spore (DNA)-mixes of 5 species.
A comparison between the number of sequence reads from a mixture of the five bacterial spores (A) and DNA extracted from an equal amount of individually extracted spore crops (B) was performed. In (C) the number of sequence reads is equalized by adjusting the amount of spore DNA on the basis of the extraction efficiency (Additional file 9A).
