# Supplementary material for: Amplicon sequencing for the quantification of spoilage microbiota in complex foods including bacterial spores
Source: Microbiome. 2015 Jul 27;3:30. doi: 10.1186/s40168-015-0096-3 (PMC4515881; doi:10.1186/s40168-015-0096-3)
Supplement: Additional file 11: — Copy number of various Bacillus genera. Using our Geobacillus thermoglucosidans TNO 09.020 16S rRNA V5-V7-sequence (OTU 3) as a BLASTN query, we reported the 16S rRNA copy numbers in various sections of the Genbank full genome library. A) For the Bacillus genera within the Firmicutes class, the number of genera with genome sequence available), the total number of 16S rRNA copies within those strains, and the average number of 16S copies are shown. B) The number of 16S rRNA copies within complete genomes of B. cereus, B. subtilis, and Geobacillus sp. is indicated. C) For Geobacillus sp., an overview of complete and draft genomes is shown. A clear underrepresentation of the number of 16S rRNA copies within the draft genomes is visible. (PPTX 550 kb) [file 40168_2015_96_MOESM11_ESM.pptx]

## Slide 1
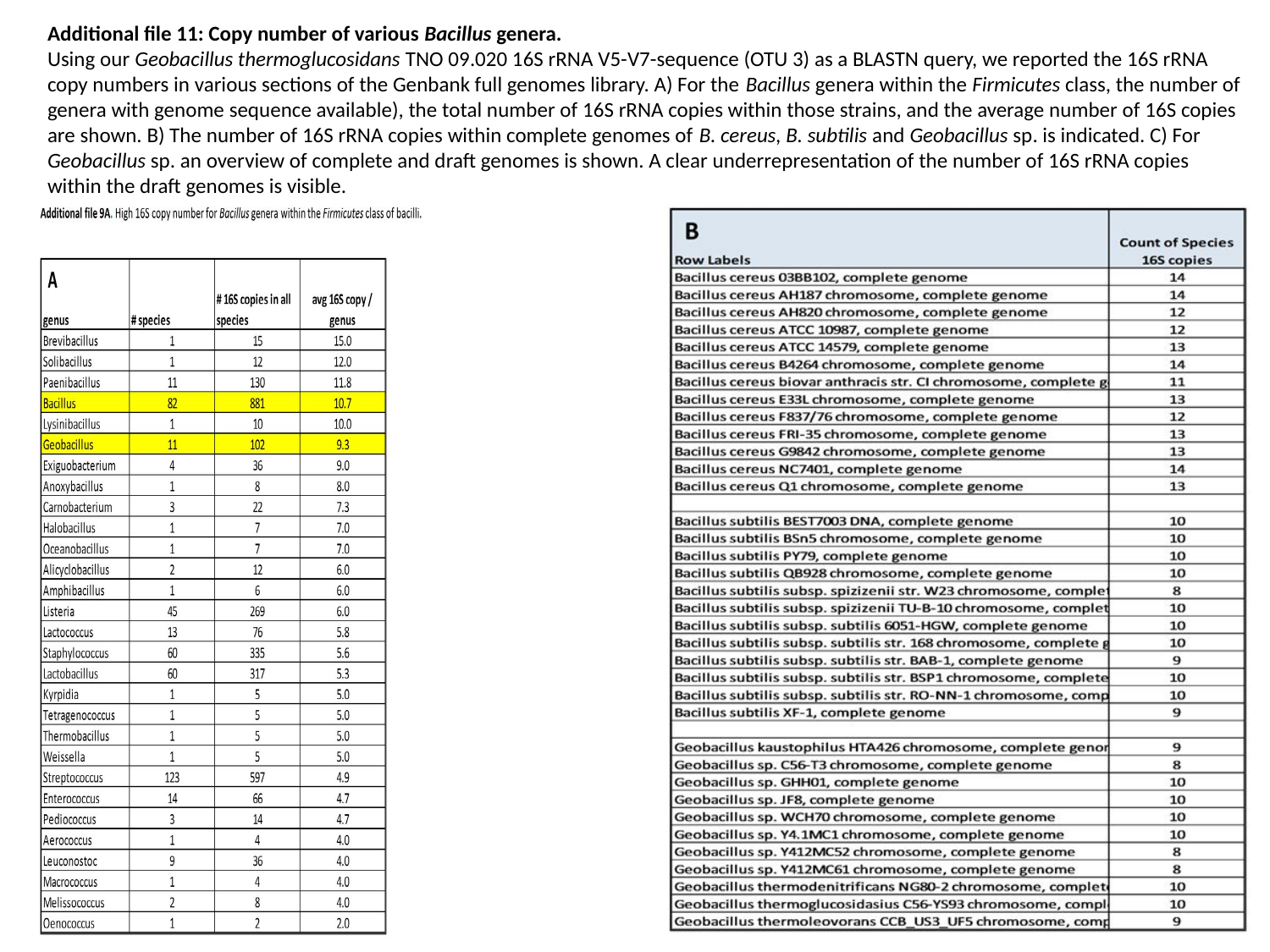

Additional file 11: Copy number of various Bacillus genera.
Using our Geobacillus thermoglucosidans TNO 09.020 16S rRNA V5-V7-sequence (OTU 3) as a BLASTN query, we reported the 16S rRNA copy numbers in various sections of the Genbank full genomes library. A) For the Bacillus genera within the Firmicutes class, the number of genera with genome sequence available), the total number of 16S rRNA copies within those strains, and the average number of 16S copies are shown. B) The number of 16S rRNA copies within complete genomes of B. cereus, B. subtilis and Geobacillus sp. is indicated. C) For Geobacillus sp. an overview of complete and draft genomes is shown. A clear underrepresentation of the number of 16S rRNA copies within the draft genomes is visible.
