# Supplementary material for: Amplicon sequencing for the quantification of spoilage microbiota in complex foods including bacterial spores
Source: Microbiome. 2015 Jul 27;3:30. doi: 10.1186/s40168-015-0096-3 (PMC4515881; doi:10.1186/s40168-015-0096-3)
Supplement: Additional file 12: — Sequence alignments of A163 and TNO-09.020 16S rRNA gene sequences indicating the presence of different 16S rRNA gene copies within the genomes of these species. A) Sequence alignment of OTU 1 and OTU 7 with the corresponding sequences of the genomic assembly fragment of B. subtilis A163 (kindly provided by Jos Boekhorst, NIZO, Ede, The Netherlands) and the sequence obtained via a 16S rRNA gene sequence typing (“PCR fragment,” Baseclear, Leiden, The Netherlands). B) Sequence alignment of OTU 3 and OTU 5 with the corresponding sequences of the genome sequence of G. thermoglucosidans TNO 09.020. (PPTX 3612 kb) [file 40168_2015_96_MOESM12_ESM.pptx]

## Slide 1
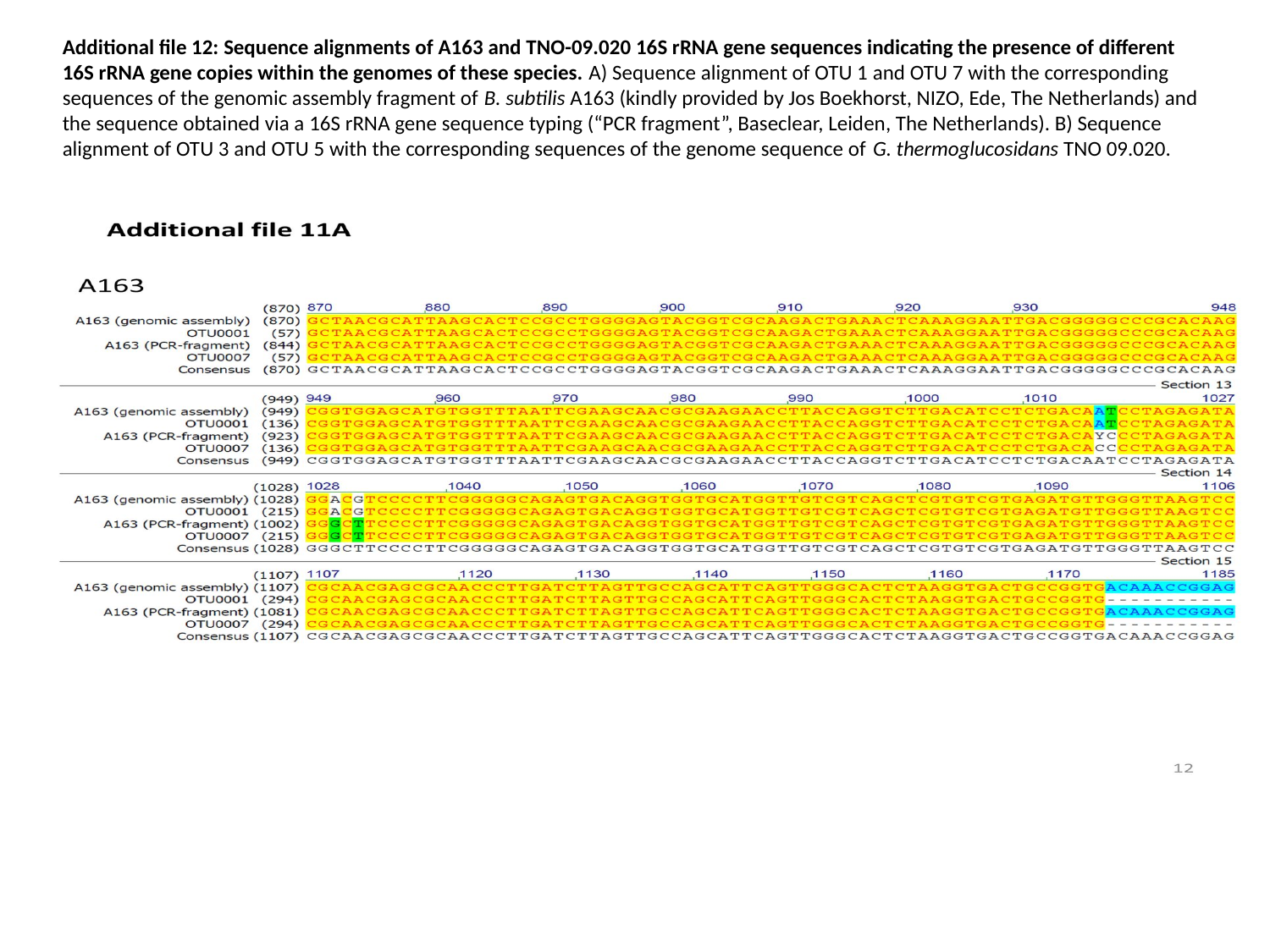

Additional file 12: Sequence alignments of A163 and TNO-09.020 16S rRNA gene sequences indicating the presence of different 16S rRNA gene copies within the genomes of these species. A) Sequence alignment of OTU 1 and OTU 7 with the corresponding sequences of the genomic assembly fragment of B. subtilis A163 (kindly provided by Jos Boekhorst, NIZO, Ede, The Netherlands) and the sequence obtained via a 16S rRNA gene sequence typing (“PCR fragment”, Baseclear, Leiden, The Netherlands). B) Sequence alignment of OTU 3 and OTU 5 with the corresponding sequences of the genome sequence of G. thermoglucosidans TNO 09.020.

## Slide 2
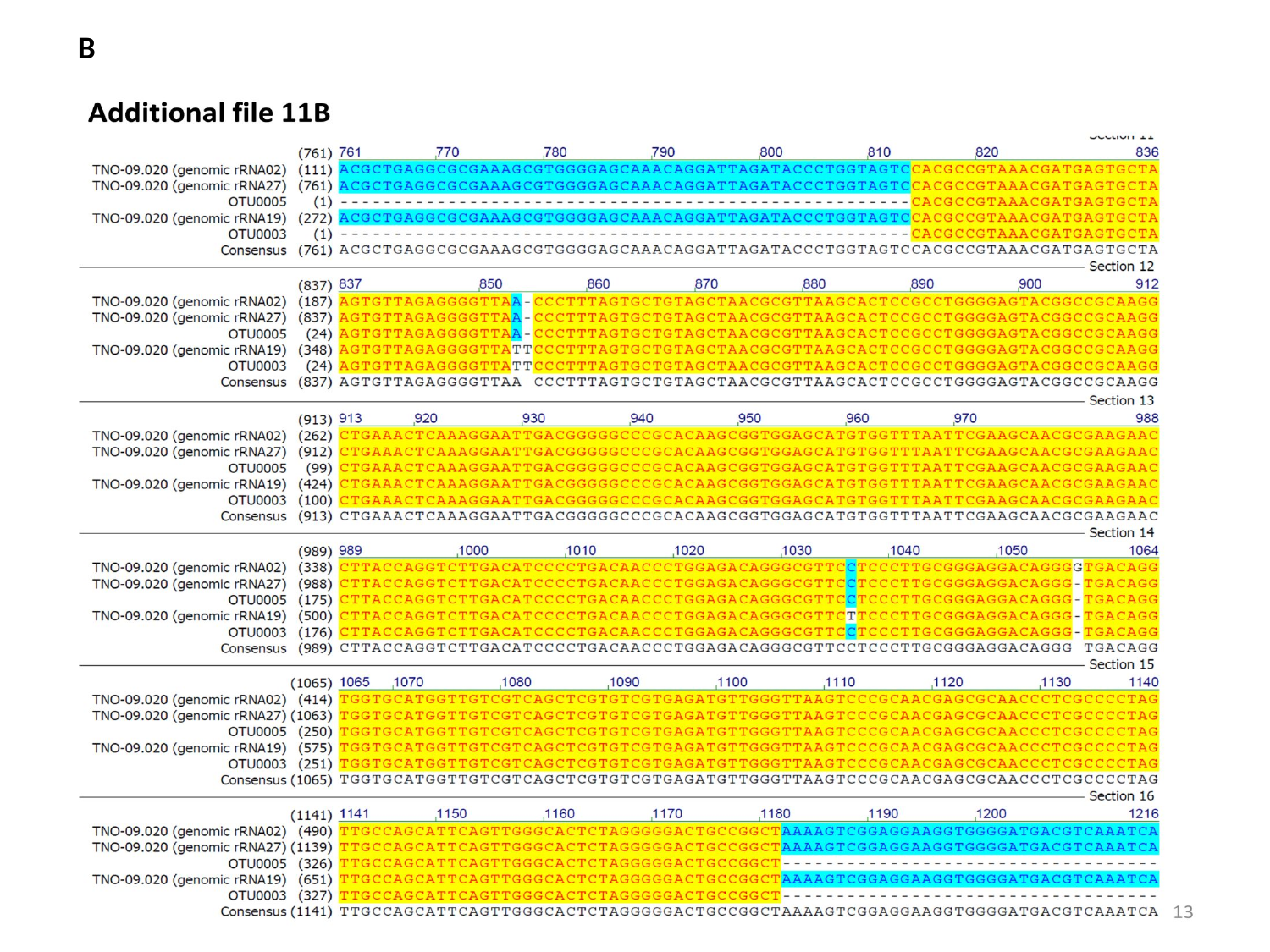

B
