# Supplementary material for: Amplicon sequencing for the quantification of spoilage microbiota in complex foods including bacterial spores
Source: Microbiome. 2015 Jul 27;3:30. doi: 10.1186/s40168-015-0096-3 (PMC4515881; doi:10.1186/s40168-015-0096-3)
Supplement: Additional file 13: — Detailed sampling scheme for a spore spoilage model for canned food. The scheme summarizes batches of canned food (creamy mushroom soup), serial dilutions of a spore mixture (of five known Bacillus species), serial dilutions of the spore mixture in canned food, and spore/canned food mixtures incubated at 37 or 55 °C. CFU counts were determined for each individual sample, whereas chromosomal DNA for 16S rRNA bar-coded amplicon sequencing was isolated from two or three aliquots (indicated in the column “# replicates”). (PPTX 383 kb) [file 40168_2015_96_MOESM13_ESM.pptx]

## Slide 1
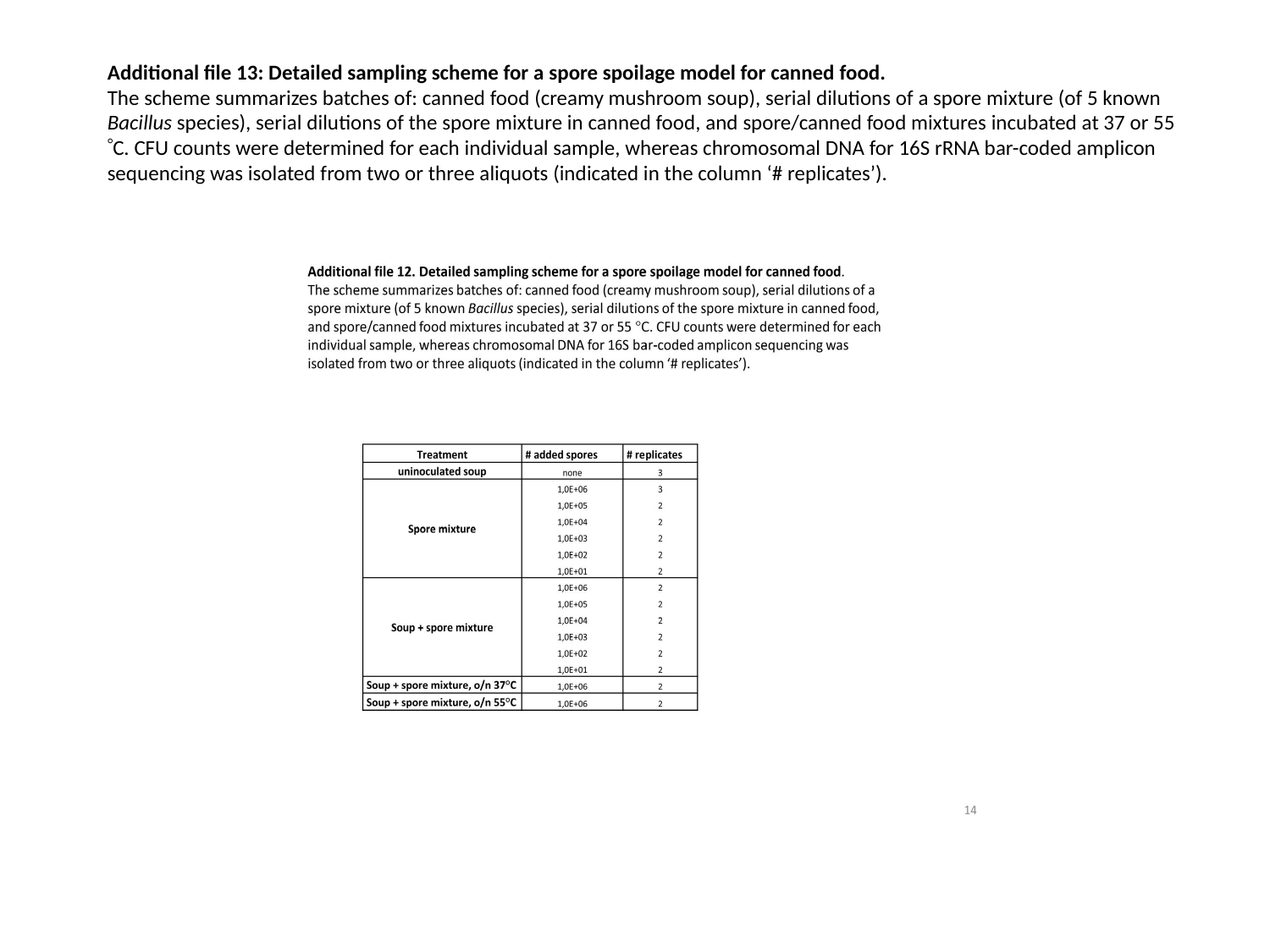

Additional file 13: Detailed sampling scheme for a spore spoilage model for canned food.
The scheme summarizes batches of: canned food (creamy mushroom soup), serial dilutions of a spore mixture (of 5 known Bacillus species), serial dilutions of the spore mixture in canned food, and spore/canned food mixtures incubated at 37 or 55 °C. CFU counts were determined for each individual sample, whereas chromosomal DNA for 16S rRNA bar-coded amplicon sequencing was isolated from two or three aliquots (indicated in the column ‘# replicates’).
