# Supplementary material for: Amplicon sequencing for the quantification of spoilage microbiota in complex foods including bacterial spores
Source: Microbiome. 2015 Jul 27;3:30. doi: 10.1186/s40168-015-0096-3 (PMC4515881; doi:10.1186/s40168-015-0096-3)
Supplement: Additional file 14: — Sampling scheme for spoilage of RTE meals in the absence/presence of food preservatives. Each sample is indicated by a number. CFU counts were determined for each individual sample, whereas chromosomal DNA for 16S rRNA bar-coded amplicon sequencing was isolated from two aliquots of selected individual samples (indicated in bold) to serve as biological duplicates. Time is shown as number of days inoculation at 7 °C. (PPTX 335 kb) [file 40168_2015_96_MOESM14_ESM.pptx]

## Slide 1
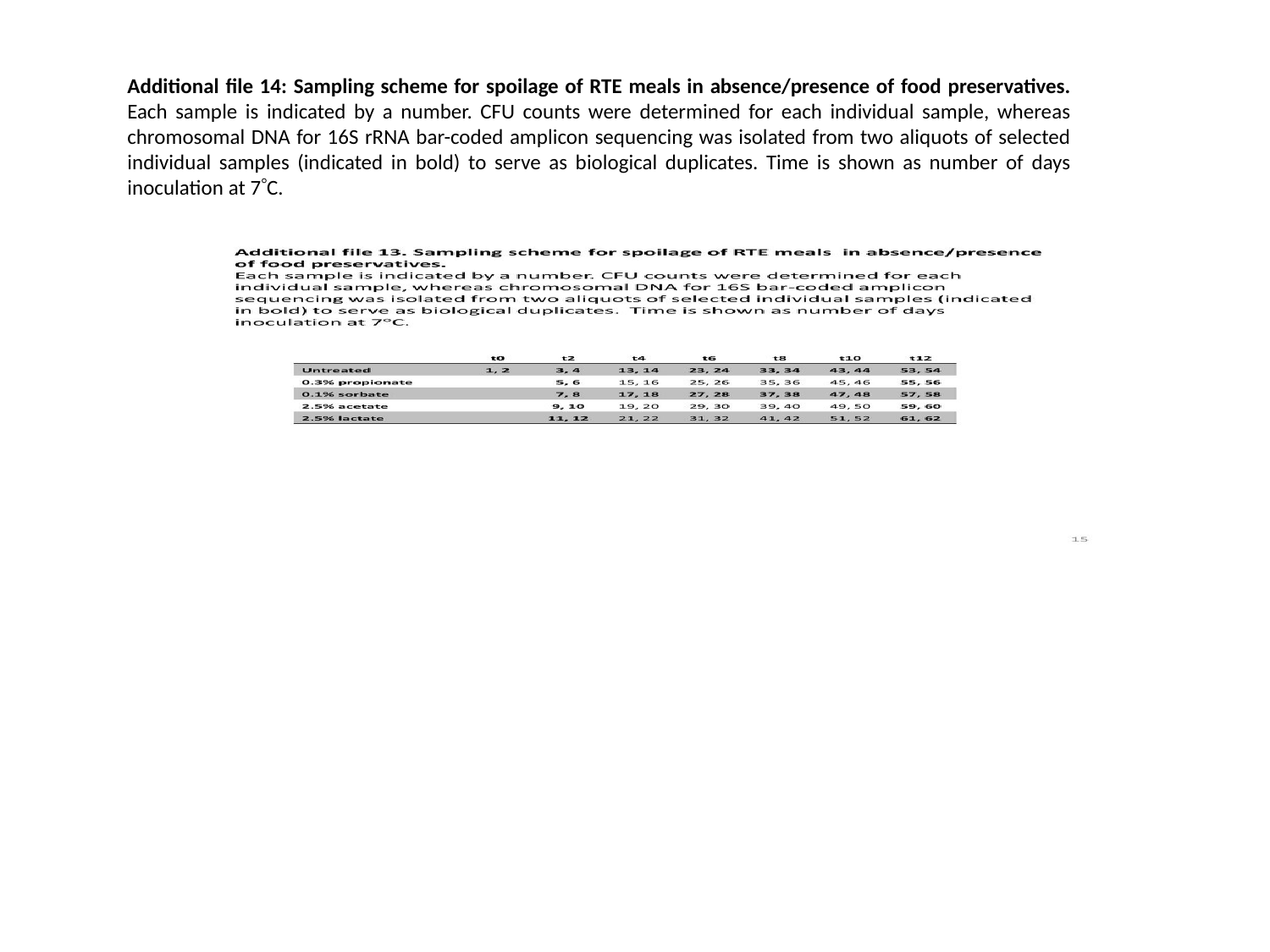

Additional file 14: Sampling scheme for spoilage of RTE meals in absence/presence of food preservatives. Each sample is indicated by a number. CFU counts were determined for each individual sample, whereas chromosomal DNA for 16S rRNA bar-coded amplicon sequencing was isolated from two aliquots of selected individual samples (indicated in bold) to serve as biological duplicates. Time is shown as number of days inoculation at 7°C.
